# Lower prevalence of obesity and nutritional imbalances in dogs fed a raw meat-based diet (RMBD) compared to a commercial complete diet

**DOI:** 10.1186/s12917-026-05283-4

**Published:** 2026-02-06

**Authors:** Lara M. von Lindeiner, Simon F. Müller, Elisabeth Müller, Romy M. Heilmann, Ingrid Vervuert

**Affiliations:** 1 Institute for Animal Nutrition, Nutritional Damage and Dietetics, Faculty of Veterinary Medicine, Leipzig University, An den Tierkliniken 9, 04103 Leipzig, Germany; 2 Department for Small Animals, Faculty of Veterinary Medicine, Leipzig University, An den Tierkliniken 23, 04103 Leipzig, Germany; 3 LABOKLIN GmbH & Co. KG, Steubenstr. 4, 97688 Bad Kissingen, Germany

**Keywords:** Canine nutrition, Body condition score (BCS), Energy, Minerals, Feeding trends, Canine health

## Abstract

**Background:**

Canine obesity is a significant health concern in spite of current feeding trends claiming healthier diets. Among these trends, raw meat-based diets (RMBD) have gained in popularity. Scientific evidence supporting these claims is limited, and nutritional imbalances are of concern in RMBD-fed dogs. This study aimed to assess the impact of RMBD-feeding on body condition in dogs in comparison to feeding a commercial complete diet (CD). A total of 104 healthy dogs were included, of which 51 dogs (age median: 4.1 years, interquartile range [IQR]: 3.2–5.9 years; body weight (BW) median: 24.8 kg, IQR: 16.3–31.9 kg) were fed an RMBD and 53 dogs (age median: 4.8 years, IQR: 3.3–5.8 years; BW median: 25.4 kg, IQR: 18.9–27.7 kg) received a CD, both for at least 12 months prior to enrollment. Enrolled dogs underwent two clinical evaluations 3–5 months apart. During these evaluations, blood, urine, and fecal samples were collected, and the patient and diet history was obtained.

**Results:**

RMBD-fed dogs had lower body condition scores (BCS; median: 5, IQR: 4–5) compared to CD-fed dogs (median: 6, IQR: 5–7, *P* < 0.001). Energy intake was lower with RMBD rations (median coverage of the daily recommended metabolizable energy [ME]: 89%) compared to CD rations (median coverage of the daily recommended ME: 102%, *P* = 0.015). Coverage of the daily energy intake was inversely correlated with BCS. In RMBD rations, the calcium (Ca): phosphorus (P) ratio (median: 1.0), failed to meet the recommended ratio of 1.4 and was lower than with CD rations (median: 1.4, P < 0.001); estimated intakes of Ca (*P* < 0.001), P (*P* < 0.001), sodium (*P* < 0.001) and magnesium (*P* = 0.004) were lower than in CD rations but close to the recommendations. Estimated intakes of Ca, P, and sodium in CD rations were at least twice the recommended amounts. Blood serum analysis revealed lower serum iodine (*P* = 0.001), copper (*P* = 0.005), zinc (*P* < 0.001), and manganese (*P* = 0.035) concentrations in RMBD-fed dogs than in CD-fed dogs.

**Conclusion:**

While RMBD-feeding might offer the advantage of a leaner body condition, concerns about nutritional imbalances warrant further investigation, even though RMBD-fed dogs do not show clinical signs of nutrient deficiencies.

**Supplementary Information:**

The online version contains supplementary material available at 10.1186/s12917-026-05283-4.

## Background

While feeding trends proposed to promote a healthier diet for dogs have gained popularity, obesity is still a growing problem in the canine population of the Western world. Feeding trends are largely influenced by cultural and social factors that determine diet schedules in people, including pet dog owners [1]. As dogs are increasingly seen as family members, nutritional trends in people have inevitably expanded to influence canine nutrition [1]. Current feeding trends in dogs include vegan or vegetarian diets and minimally-processed biologically appropriate raw food (BARF) or raw meat-based diets (RMBD) [2]. RMBDs date back to the 1990 s and follow the concept of “a raw meat-based diet as wolves eat it” [3]. The prevalence of RMBD feeding of dogs ranges from 25% worldwide to 60% reported for the Netherlands [4–6].

Dog owners who elect to feed an RMBD perceive this dietary strategy to be more natural and as the most appropriate choice for the canine species [7], promoting health benefits in their dogs, including improved dental health, skin barrier, and fecal consistency with reduced fecal output [2, 4]. However, only a few studies provide scientific evidence for enhanced health status to support these anecdotal reports or perceptions [8–10]. In contrast, nutritionists emphasize the risk of nutritional imbalances from RMBD feeding. An unbalanced RMBD can cause an over- or undersupply of nutrients in dogs. It has been shown that RMBDs are often deficient in calcium (Ca), iodine (I), zinc (Zn), copper (Cu), and vitamins A and D, whereas the supply of protein and phosphate can be excessive [11].

Despite the increasing awareness of dog owners for the importance of a healthy canine diet, overweight and obesity are common conditions in dogs [12, 13]. Chronic oversupply of energy leads to – among other consequences – weight gain and, eventually, obesity [14], highlighting the importance of an adequate energy intake. Dogs are categorized as “obese” when body weight exceeds the calculated ideal (lean) body weight by more than 30% [15] and as “overweight” with an increased body weight below that cut-off. The prevalence of overweight and obesity in dogs is on the rise, having ranged from 20–40% in the UK, US, and Denmark in the 1990 s [16–18] compared to more recent studies reporting a prevalence of 20–59% depending on the country where the studies were pursued (Scotland, Netherlands, UK, Spain, Brazil, Australia, and Germany) [12, 19–24]. Being overweight or obese can have several adverse health effects, leading to chronic low-grade inflammation [25–27] and a shorter lifespan of affected dogs [28].

While obesity has been studied in the general dog population [12, 28], limited data are available comparing the body condition score (BCS) to assess health and overweight status between healthy dogs fed an RMBD and healthy dogs receiving a commercial complete diet (CD) [10].

Therefore, the aim of the study was to assess the impact of RMBD-feeding on the body condition in healthy dogs in comparison to feeding a CD. We hypothesized that dogs fed exclusively an RMBD are less frequently affected by overweight or obesity than dogs receiving a CD. Thus, the study aimed to evaluate the impact of ration composition together with the physical activity level on the overall health and nutritional status in a population of healthy dogs fed either an RMBD or a CD.

## Results

### Study population characteristics

A total of 104 healthy dogs were included in the data analysis, of which 51 dogs received an RMBD and 53 dogs a CD. The RMBD group (n = 51) was comprised of 41 pure-bred dogs (27 different breeds) and 10 mixed-breed dogs, weighing between 7.4 kg and 37.5 kg. The CD group (n = 53) included 34 pure-bred dogs (23 different breeds) and 19 mixed-breed dogs, with body weights ranging from 8.3–37.2 kg (Add. file 1).

Age (*P* = 0.530), sex distribution (*P* = 0.442), and reproductive status (*P* = 0.151) did not differ between the two feeding groups of dogs (Table 1), but significantly more mixed-breed dogs were CD-fed than RMBD-fed (*P* = 0.036, effect size [ES] = 0.21). Anti-rabies vaccination status differed significantly (*P* = 0.026) between both feeding groups of dogs, but not the vaccination status for the pentavalent canine vaccine (*P* = 0.081) (Table 1).

**Table 1:** Population characteristics and physical examination findings in n = 104 healthy dogs included in the study

Variables	RMBD-fed dogs	CD-fed dogs	P*
n	**51**	**53**	
***Patient characteristics***			
Age [years]^#^	4.1 (3.2–5.9)	4.8 (3.3–5.8)	0.530
Sex, male (neutered)/female (spayed)	25 (11)/26 (6)	22 (14)/31 (11)	0.442 (0.151)
Breed			
Pure-bred/mixed-breed	41 (80)/10 (20)	34 (64)/19 (36)	**0.036**
Body weight [kg]^#^	24.8 (16.3–31.9)	25.4 (18.9–27.7)	0.938
BCS [on 9-point scale]^#^	5 (4–6)	6 (5–7)	** < 0.001**
***Environmental and lifestyle factors***			
Deworming status			** < 0.001**
Regular empirical deworming	25 (49%)	45 (85%)	
Regular deworming based on flotation analysis	23 (45%)	7 (13%)	
No deworming	3 (6%)	1 (2%)	
Vaccination			
Vaccination against rabies			**0.026**
Basic immunization	3 (6%)	–	
Regular booster vaccination (q1-3 years)	46 (90%)	53 (100%)	
No vaccination	2 (4%)	–	
Pentavalent^&^ vaccination			0.081
Basic immunization	6 (12%)	2 (4%)	
Regular booster vaccination (1–3 years)	43 (84%)	51 (96%)	
No vaccination	2 (4%)	–	
***Physical examination findings***			
BT [°C]^$^	38.1 (37.9–38.5)	38.4 (38.2–38.7)	**0.001**
BT [°F]^$^	100.6 (100.2–101.3)	101.1 (100.8–101.7)	**0.001**
HR [heart beats/min]^#^	96 (80–104)	92 (84–104)	0.787
RR [breaths/min]^#^	24 (20–27)	24 (20–28)	0.358
Dental calculus^§^			0.362
None present	40 (78%)	39 (74%)	
Low-grade	10 (19%)	14 (26%)	
Medium-grade	1 (2%)	–	

Findings on physical examination at timepoint (TP) 1 are summarized as medians and interquartile ranges [IQR] or counts (n) and proportions (%). Data were similar to those upon TP2; therefore, these descriptive statistics are not shown. *P-values refer to the comparison of the two feeding groups of dogs. ^#^Non-parametric comparison using the Mann–Whitney U test. RMBD = raw meat-based diet; CD = commercial diet; BT = rectal body temperature; HR = heart rate; RR = respiratory rate. ^&^Vaccination against distemper, hepatitis, parvo, and parainfluenza virus and *Leptospira* spp.. ^$^Parametric comparison using an unpaired t test. ^§^Categorized based on Ramfjord 1959 [29]

Cohabitation of the study dogs with other pet animals did not differ between both feeding groups of dogs. About half of the CD-fed dogs (n = 26; 49%) lived in a multi-pet household; nine of these dogs were housed with at least one cat, and 17 of the dogs were in a multi-dog home. All housemate pets were also fed a CD. Nearly two-thirds (n = 32; 63%) of the RMBD-fed dogs lived in a multi-pet household, of which 27 dogs lived in multi-dog homes where the partner dogs were either fed an RMBD (25 households) or a CD (2 households). In 3 multi-pet-homes, housemate dogs and cats were fed a CD, while in 2 households, the partner cats received a CD.

The two most important motivations for dog owners electing to feed an RMBD to their dog were selecting a diet that is considered to be “natural” (44%) and addressing an allergy or intolerance to commercial feed as presumed by the owners (21%). Other less frequently reported motivations were improved fecal quality or reduced fecal output, a recommendation from the breeder or other dog owners, perceived improvement in coat or hair quality, the ability to influence the feed composition, and skepticism towards commercial feed. RMBD rations were calculated mostly by free online RMBD calculator tools (49%) or manually by the owners (36%), whereas owners of CD-fed dogs referred mainly to the labelled feeding recommendation provided by the manufacturer (62%) to determine the amount of food offered.

### Physical examination findings

All dogs were assessed as clinically healthy. Dental health of the dogs did not differ between both feeding groups of dogs (*P* = 0.362). The majority of dogs (99%) showed no dental calculus or it was classified as low grade (Table 1). Faciocranial abnormalities, such as an underbite or broken tooth tips, were seen in 5 RMBD-fed dogs (10%) and 7 CD-fed dogs (13%). One RMBD-fed dog had a small umbilical hernia without clinical consequences.

### Body constitution and activity level

**Fig. 1:**
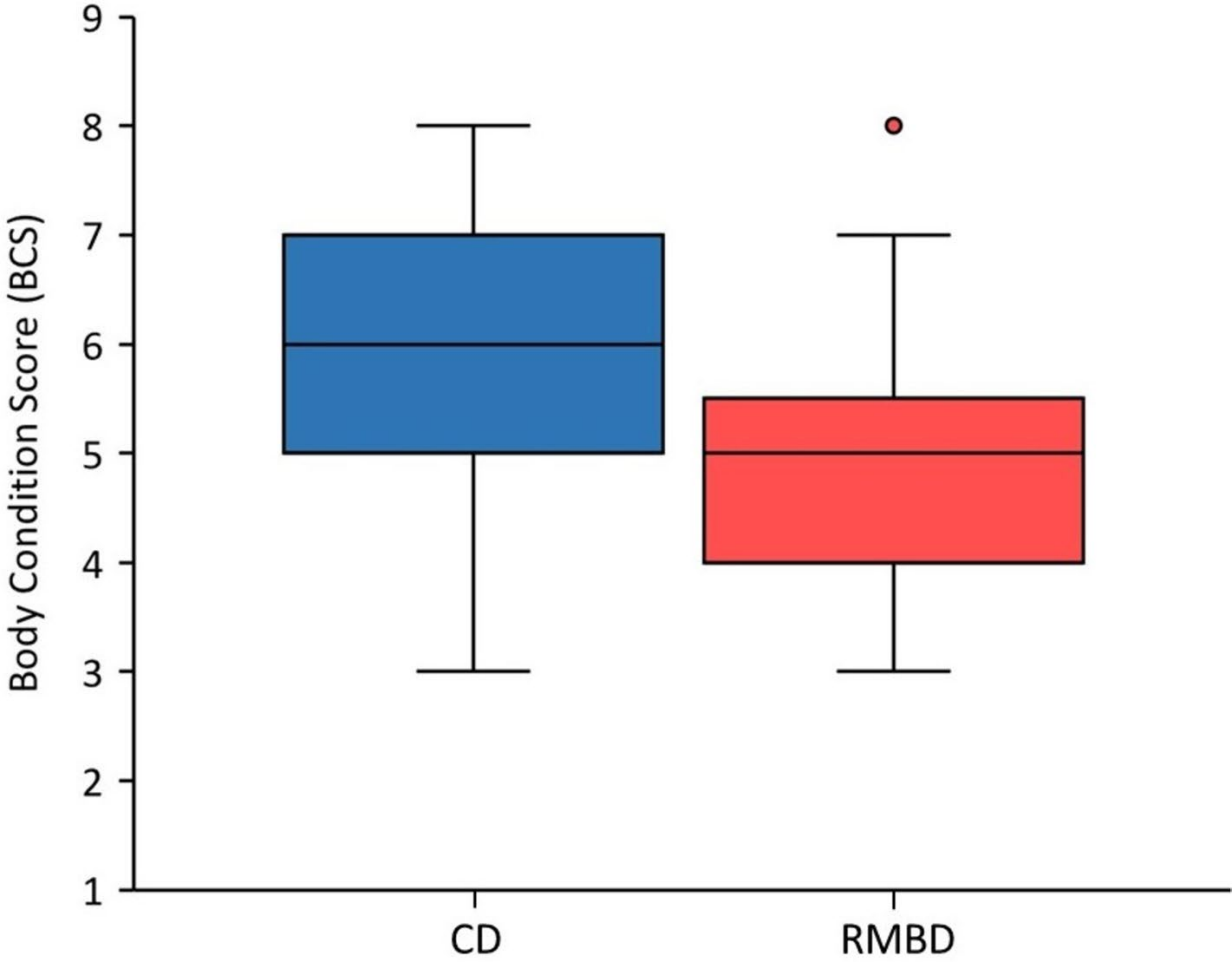
Body condition of the dogs included in the study. CD-fed dogs had significantly higher body condition scores ([BCS]; median: 6, interquartile range [IQR]: 5–7) than RMBD-fed dogs (median: 5, IQR: 4–6; *P* < 0.001). The length of the box represents the IQR, the horizontal line of the box is the median, and the whiskers represent 1.5-times the IQR below the 25th quartile and above the 75th quartile; the dot represents an outlier (ITF). RMBD = raw meat-based diet; CD = commercial diet

Muscle condition scores (MCS) were assessed as normal for all dogs, and there was no significant difference in the distribution of body weights between the two feeding groups of dogs (*P* = 0.938; Table 1). However, BCS was significantly lower in RMBD-fed dogs than in CD-fed dogs (*P* < 0.001, ES = 0.45; Table 1; Fig. 1). Owner assessment of their dog's BCS did not differ from the veterinarian’s assessment in RMBD-fed dogs (for both median: 5, IQR: 4–5; *P* = 0.349,). In contrast, BCS was assessed as significantly lower by the owners as compared to the veterinarian in CD-fed dogs (median: 5, IQR: 5–5 vs. median: 6, IQR: 5–7; *P* < 0.001), and both assessments were only weakly correlated (ρ = 0.34, *P* = 0.013).

Minor body weight changes were detected between TP1 and TP2 in both feeding groups of dogs (RMBD: 0%; CD: 1.3%; Add. file 2). Individual CD-fed dogs had a weight gain of 16% (moderate outlier outside the calculated outer Tukey fence [OTF]) or a weight loss of 6% (mild outlier inside the calculated outer Tukey fence [ITF]).

**Fig. 2:**
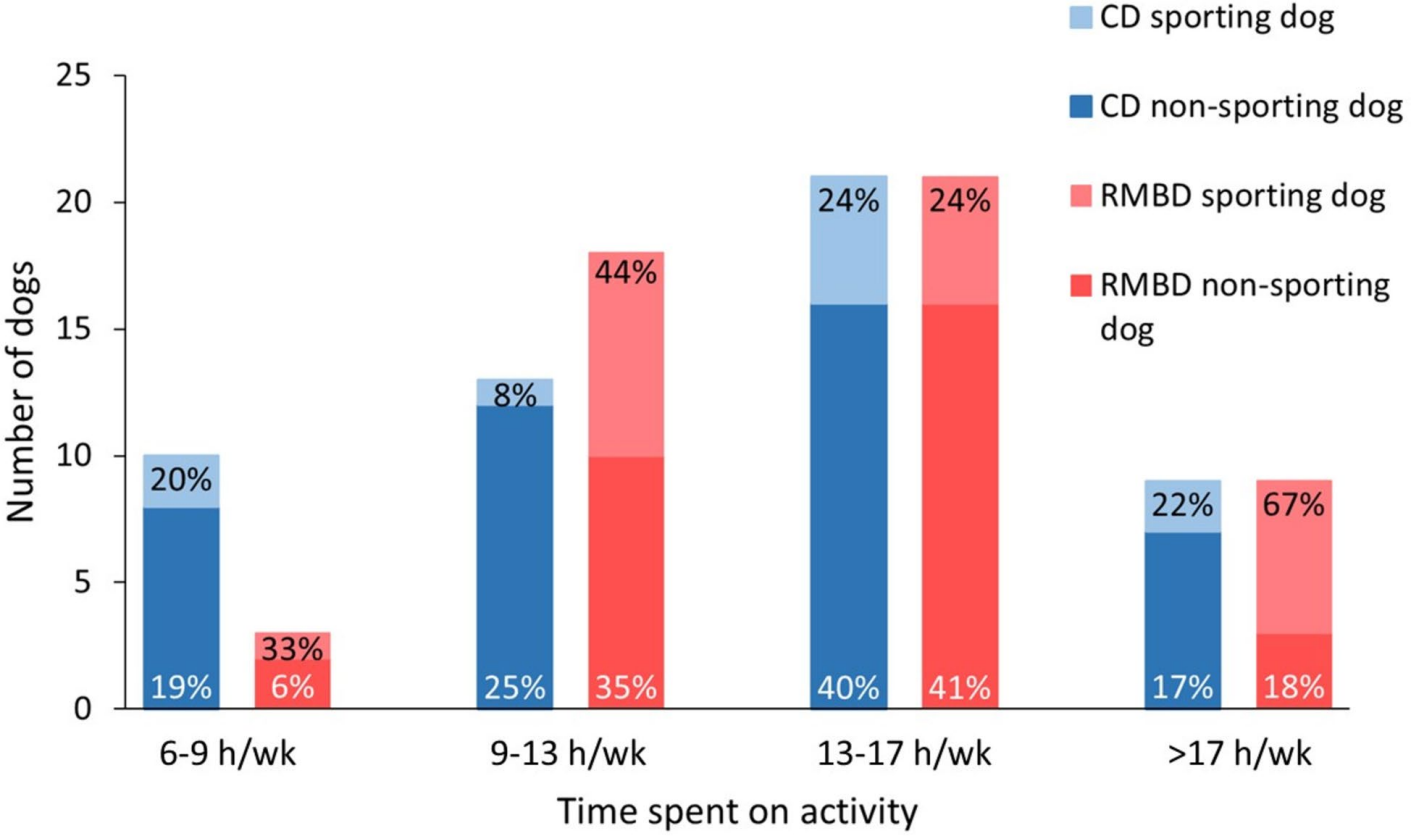
Bar chart summarizing indicators of physical activity for the dogs fed either an RMBD or CD. Shown are the numbers of dogs, stratified by their denomination as either “sporting dog” or “non-sporting dog”, subclassified based on the average time (in hours per week [h/wk]) engaged in high-energy level activities together with the owner. The percentages in white font represent the proportion of “non-sporting” dogs within each feeding group, and the percentages in black font indicate the proportion of “sporting” dogs in each feeding group. RMBD = raw meat-based diet; CD = commercial diet. CD sporting dog = CD-fed dog engaged in dog sports; CD non-sporting dog = CD-fed dog not engaged in dog sports; RMBD sporting dog = RMBD-fed dog engaged in dog sports; RMBD non-sporting dog = RMBD-fed dog not engaged in dog sports; h = hours; wk = week

Regardless of the time spent weekly on physical activity, significantly more RMBD-fed dogs engaged in regular sporting activities (n = 20, 39%) compared to CD-fed dogs (n = 10, 19%; *P* = 0.022, ES = 0.21). Less than 9 h per week (h/wk) were spent on active time with the dog by significantly more owners of CD-fed dogs (19%; n = 10) compared to RMBD-fed dogs (6%; n = 3; *P* = 0.045, ES = 0.20; Fig. 2). Despite similar proportions of dogs with very high activity levels (> 17 h/wk) in both feeding groups of dogs (RMBD: 18%, n = 9; CD: 17%, n = 9), there were more RMBD-fed dogs with very high activity levels that participated in sporting activities (67%; n = 6) compared to CD-fed dogs (22%; n = 2), but the difference did not reach statistical significance (*P* = 0.153). While the average time spent on physical activity per week was not correlated with the dog’s BCS (*P* = 0.398; Add. file 3), there was an inverse correlation between the average time spent on dog sporting activities and the dog´s BCS (ρ = −0.27, *P* = 0.006).

### Estimated energy and nutrient intake

**Fig. 3:**
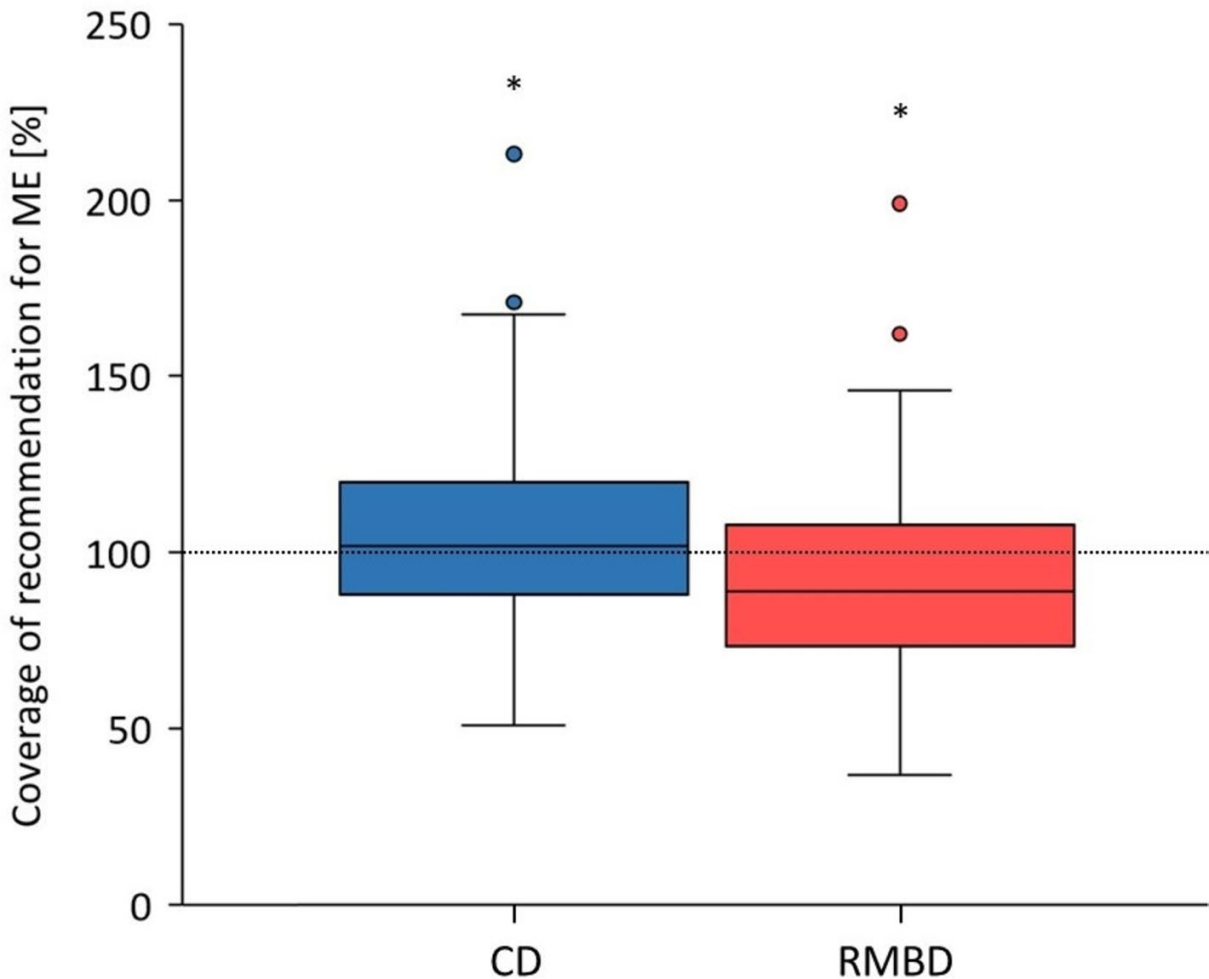
Coverage of the daily recommendation for metabolizable energy (ME; in %) in RMBD- *vs.* CD-fed dogs. Coverage of the ME recommendation was significantly lower with RMBD rations (median: 89%, interquartile range [IQR]: 74–108%) than with CD rations (median: 102%, IQR: 88–120%,*P* = 0.015, effect size [ES] = 0.24). The length of the box represents the IQR, the horizontal line of the box is the median, and the whiskers represent 1.5-times the IQR below the 25th quartile and above the 75th quartile; the dots represent outliers (ITF), and asterisks are extreme outliers (OTF). The dashed line marks a full coverage of the daily recommendation for ME intake by the NRC (100%). RMBD = raw meat-based diet; CD = commercial diet

**Fig. 4:**
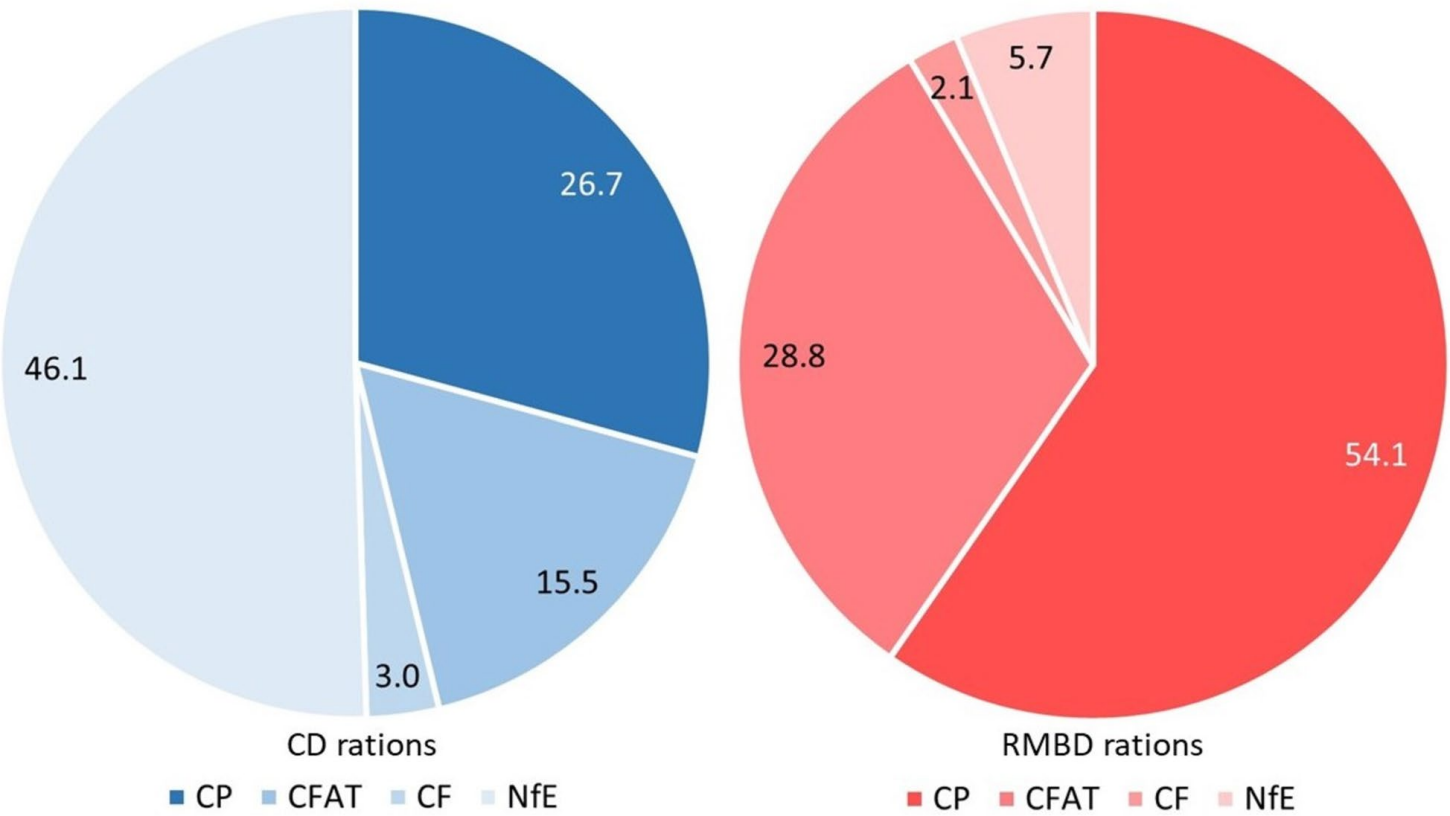
Relative qualitative energy supply (in % dry matter [DM]) in the rations of the different feeding types. CD rations (left panel): CP: median: 26.7% DM, interquartile range (IQR): 23.9–33.1% DM; CFAT: median: 15.5% DM, IQR: 13.1–19.5% DM; CF: median: 3.0% DM, IQR: 2.5–3.6% DM; NfE: median: 46.1% DM, IQR: 36.9–51.2% DM. RMBD rations (right panel): CP: median: 54.1% DM, IQR: 47.6–59.1% DM; CFAT: median: 28.8% DM, IQR: 24.5–32.9% DM; CF: median: 2.1% DM, IQR: 1.0–3.2% DM; NfE: median: 5.7% DM, IQR: 3.4–14.9%DM. CP = Crude protein; CFAT = Crude fat; CF = Crude fiber; NfE = N-free extractives

Coverage of the daily recommendation for estimated metabolizable energy (ME) was significantly lower with RMBD rations (median: 89%, IQR: 74–108%) than CD rations (median: 102%, IQR 88–120%; *P* = 0.015, ES = 0.24; Fig. 3). Individuals in both feeding groups of dogs received rations with up to 225% (RMBD, n = 1, OTF) and 233% ME (CD, n = 1, OTF). Coverage of the daily energy intake negatively correlated with BCS of the dogs (ρ = −0.249, *P* = 0.011; Add. file 4). Dogs on RMBD rations received a median of 54.1% of the estimated recommended ME provided by protein, while the main energy source was carbohydrates in CD rations (median: 46.1% DM; Fig. 4). The proportion of crude fiber (CF) in % of dry matter (DM) was significantly lower in RMBD rations (median: 2.1% DM, IQR: 1.0–3.2% DM) than in CD rations (median: 3.0% DM, IQR: 2.5–3.6% DM; *P* < 0.001, ES = 0.37). RMBD rations contained a higher proportion of crude fat (CFAT; median: 28.8% DM) than CD rations (median: 15.5% DM).

**Fig. 5:**
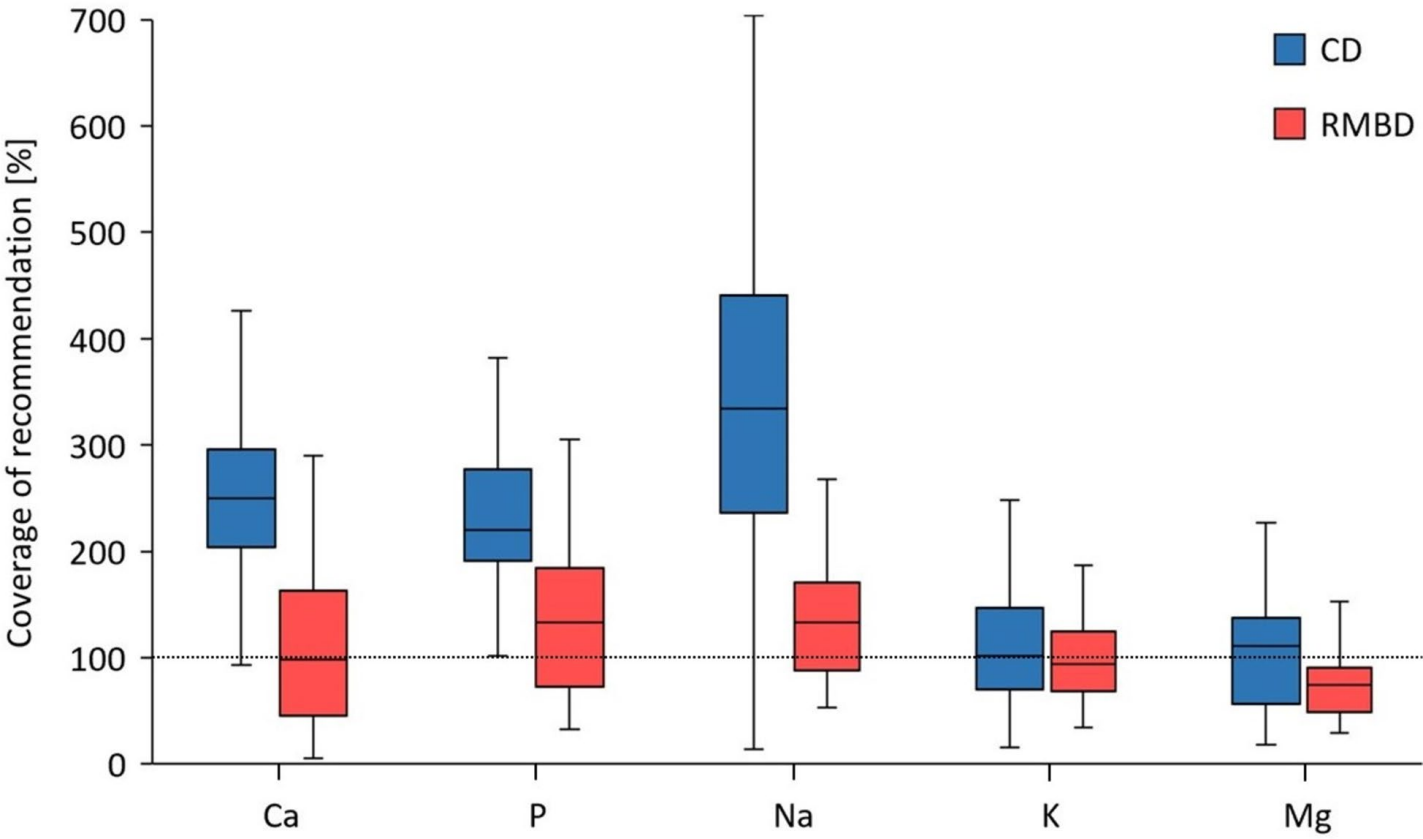
Coverage of the daily recommendation (in %) for the various major minerals in the rations. The coverage of the recommendation by the NRC was significantly higher in CD rations for Ca (median: 250%, interquartile range [IQR]: 204–296%), P (median: 220%, IQR: 192–277%), Na (median: 335%, IQR: 237–441%), and Mg (median: 111%, IQR: 57–138%) than in RMBD rations (Ca: median: 98%, IQR: 46–163%, *P* < 0.001, effect size [ES] = 0.62; P: median: 133%, IQR: 73–184%, *P* < 0.001, ES = 0.53; Na: median: 133%, IQR: 88–171%, *P* < 0.001, ES = 0.74; and Mg: median: 75%, IQR: 49–91%, *P* = 0.004, ES = 0.30). Coverage of the recommendation for K did not differ between CD rations (median: 102%, IQR: 70–147%) and RMBD rations (median: 94%, IQR: 69–125%, *P* = 0.495). The length of the box represents the IQR, the horizontal line of the box is the median, and the whiskers represent 1.5-times the IQR below the 25th quartile and above the 75th quartile. Outliers are not shown. The dashed line marks a full coverage of the daily recommendations by the NRC (100%). RMBD = raw meat-based diet; CD = commercial diet

**Fig. 6:**
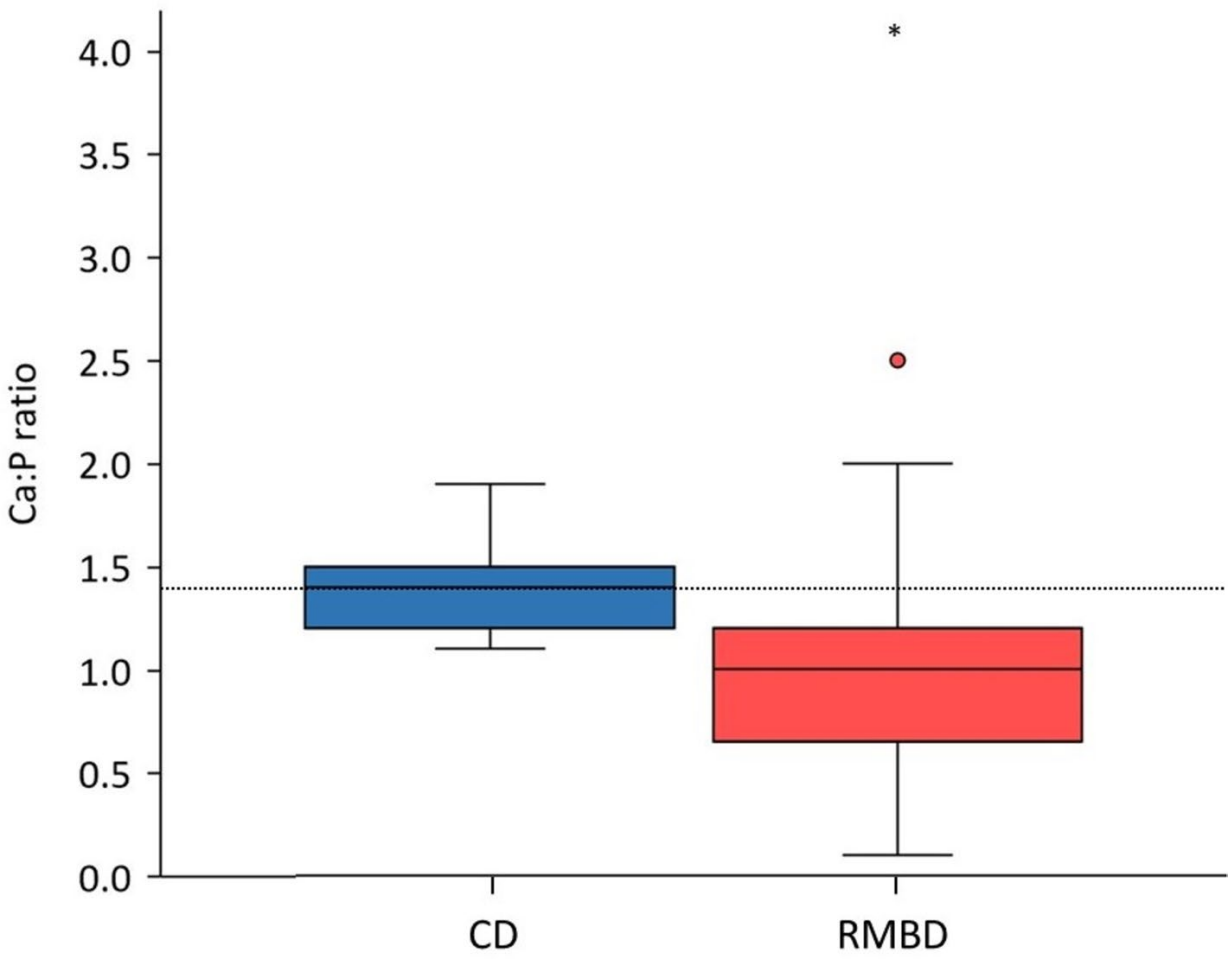
Ca:P ratio in the rations of the two feeding groups of dogs. The Ca:P ratio was significantly lower in RMBD rations (median: 1.0, interquartile range [IQR]: 0.7–1.2) than in CD rations (median: 1.4, IQR: 1.2–1.5; *P* < 0.001, effect size [ES] = 0.48). The length of the box represents the IQR, the horizontal line of the box is the median, and the whiskers represent 1.5-times the IQR below the 25th quartile and above the 75th quartile; the dot represents an outlier (ITF), and the asterisk represents an extreme outlier (OTF). The dashed line marks a recommended Ca:P ratio of 1.4. RMBD = raw meat-based diet; CD = commercial diet

Estimated coverages of the National Research Council (NRC) recommendation for Ca, P, sodium (Na), and magnesium (Mg) were significantly higher in CD than in RMBD rations (Fig. 5). In CD rations, calculated intake for Ca, P, and Na ranged from 2.3- to 3.4-fold NRC recommendation coverage. In contrast, these estimated intakes were close to the recommendations in RMBD rations. NRC recommendation coverage for potassium (K) did not differ between CD and RMBD rations (Fig. 5). In RMBD rations, the Ca:P ratio did not meet the NRC recommendation of 1.4 and was significantly lower (median: 1.0) than in CD rations (median: 1.4, *P* < 0.001; Fig. 6). Two RMBD-fed outliers received rations with a Ca:P ratio of 2.5 (ITF) and 4.1 (OTF).

**Fig. 7:**
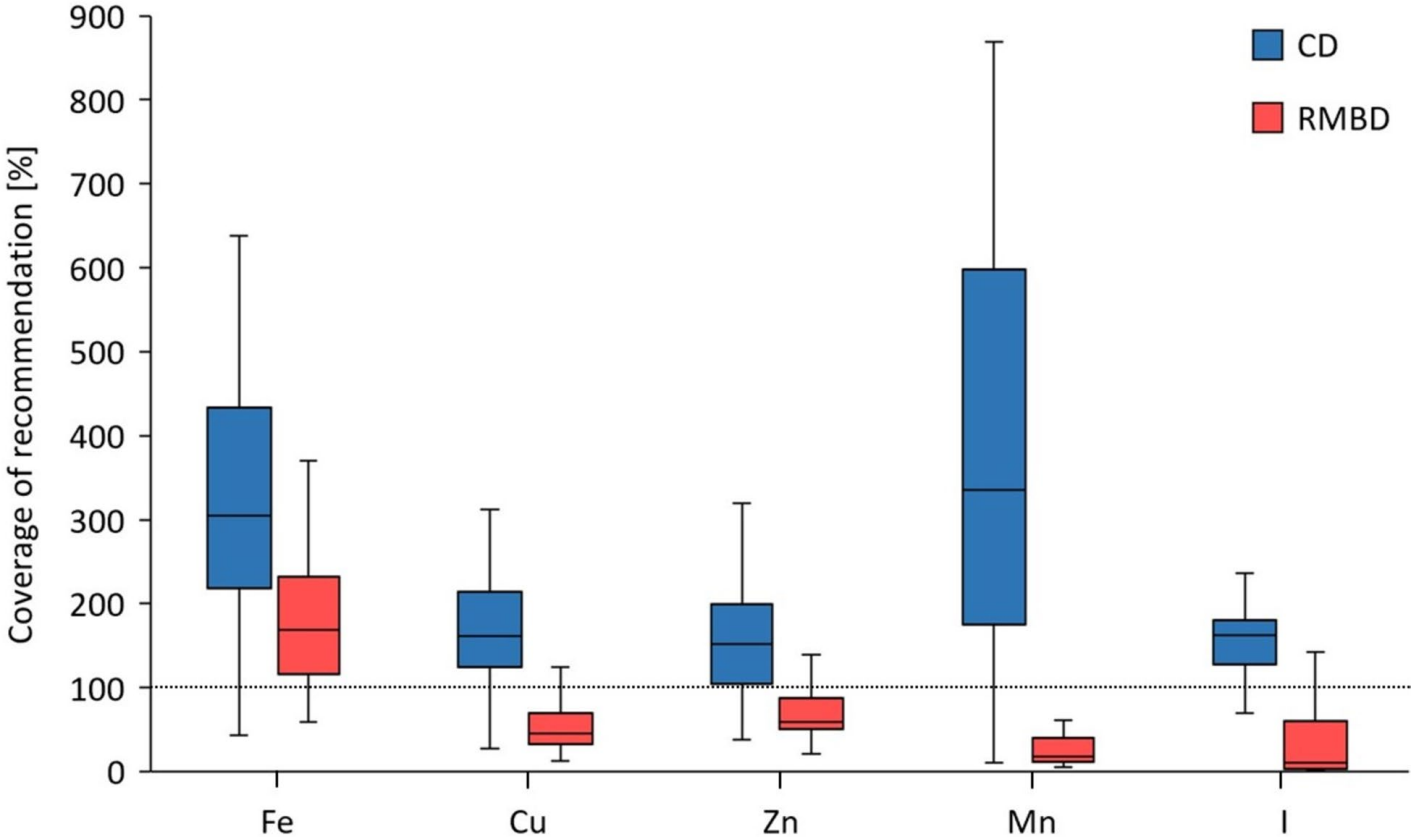
Coverage of the daily recommendation (in %) for the various trace elements in the rations. The coverage of the recommendation was significantly higher in CD rations for Fe (median: 305%, interquartile range [IQR]: 218–433%), Cu (median: 161, IQR: 124–215%), Zn (median: 152%, IQR: 104–199%), Mn (median: 336%, IQR: 175–599%), and I (median: 162%, IQR: 123–181%) than in RMBD rations (Fe: median: 169%, IQR: 117–232%, *P* < 0.001, effect size [ES] = 0.48; Cu: median: 46%, IQR: 33–70%, *P* < 0.001, ES = 0.67; Zn: median: 60%, IQR: 51–88%, *P* < 0.001, ES = 0.63; Mn: median: 18%, IQR: 12–40%, *p* < 0.001, ES = 0.71; and sI: median: 13%, IQR: 11–60%, *P* < 0.001, ES = 0.66). The length of the box represents the IQR, the horizontal line of the box is the median, and the whiskers represent 1.5-times the IQR below the 25th quartile and above the 75th quartile. Outliers are not shown. The dashed line marks a full coverage of the daily recommendations by the NRC (100%). RMBD = raw meat-based diet; CD = commercial diet

**Fig. 8:**
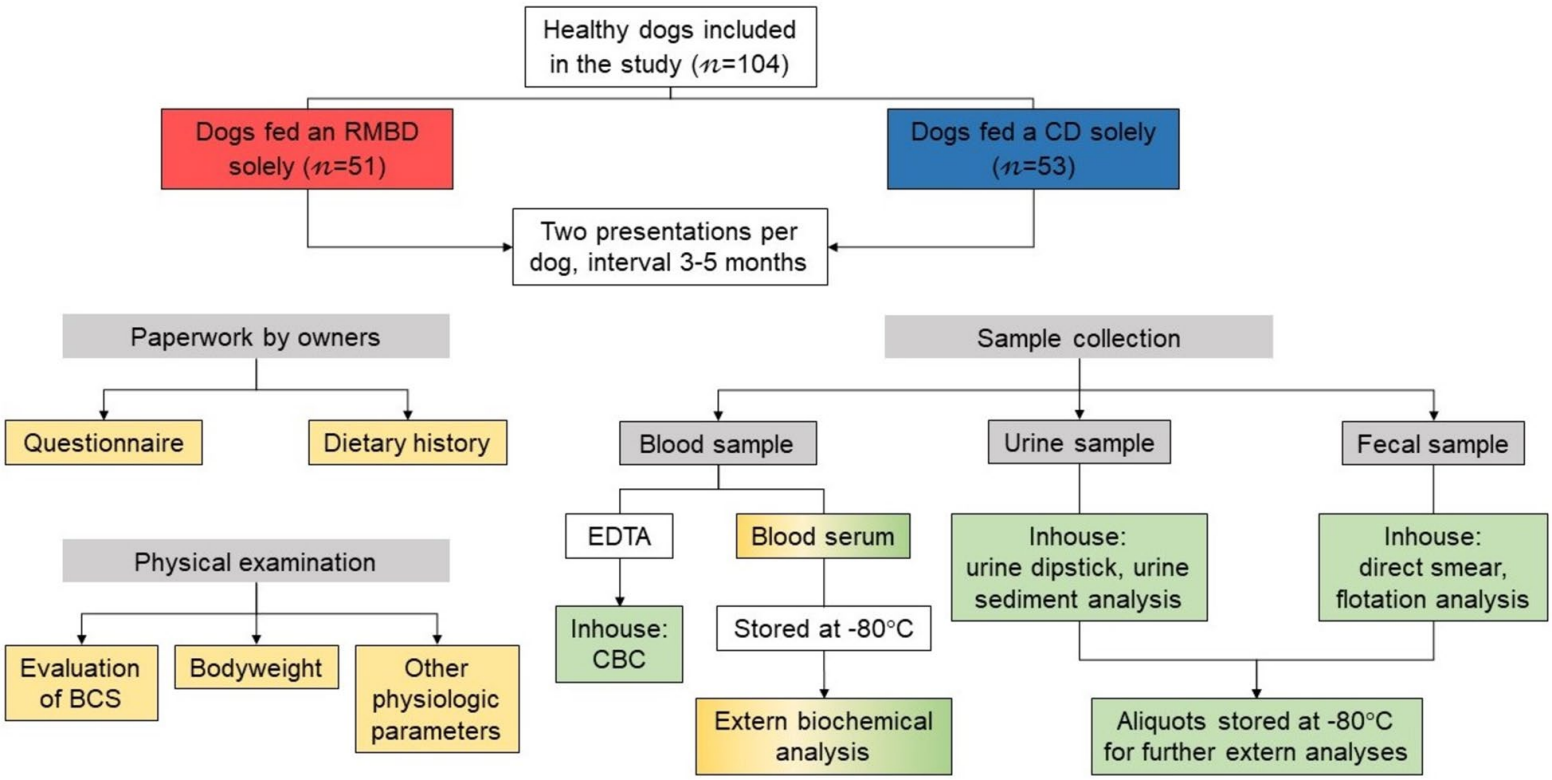
Flowchart summarizing the study design. Results of the areas in yellow color are included in this report, results of the areas in green are presented in the companion article (reference to paper 2). RMBD = raw meat-based diet; CD = commercial diet; BCS = body condition score; CBC = complete blood cell count

The daily NRC recommendations were met for calculated iron (Fe) intake in both feeding groups of dogs. Fe, copper (Cu), zinc (Zn), manganese (Mn), and iodine (I) intakes were significantly lower in RMBD than CD rations (all *P* < 0.001; ES for Fe = 0.48, Cu = 0.,67, Zn = 0.63, Mn = 0.71, and I = 0.66; Fig. 7). Daily NRC recommendations for Cu and Zn intake were not met, and the median NRC recommendation coverage for Mn and I were less than 20% in RMBD rations (fig. 8).

Daily vitamin D intakes were significantly lower with RMBD rations (median: 18% of the NRC recommendation, IQR: 8–66%) than with CD rations (median: 174%, IQR: 141–245%, *P* < 0.001, ES = 0.75). Similarly, the intake of vitamin E was significantly lower for RMBD rations (median: 41% of the NRC recommendation, IQR: 26–107%) compared to CD rations (median: 336%, IQR: 190–651%, *P* < 0.001, ES = 0.68). In contrast, vitamin A intakes did not differ between the two feeding groups of dogs (*P* = 0.668), with a 0.4- to 4.6-fold NRC recommendation coverage for vitamin A (median: 331%, IQR: 41–459%) with RMBD rations and a 1.9- to 3.1-fold NRC recommendation coverage (median: 223%, IQR: 191–311%) with CD rations.

### Blood biochemistry variables

Blood serum biochemistry analysis and complete blood cell (CBC) count confirmed the overall health status of the dogs; with full details of these results reported in the companion article (reference to paper 2). Blood serum concentrations of the major minerals (except for total Ca), Fe, and vitamins D and A did not differ between the two feeding groups of dogs (Table 2). Blood serum concentrations of total Ca were significantly lower in RMBD- vs. CD-fed dogs (*P*_*corr*_ = 0.030), but all dogs had blood serum concentrations of all major minerals within the respective normal reference interval (RI).

**Table 2:** Results of mineral and vitamin analyses in blood serum samples from 104 healthy dogs included in the study

Parameter	RMBD-fed dogs	CD-fed dogs	RI	*P*^§^	*P*_*corr*_
n	**51**	**53**			
***Major minerals***					
Total Ca [mmol/L]^&^	2.5 (2.5–2.6)	2.6 (2.5–2.6)	2.3–3.0	**0.006**	**0.030**
P [mmol/L]^$^	1.2 (1.1–1.3)	1.2 (1.1–1.3)	0.7–1.6	0.420	1
Na [mmol/L]^$^	148 (147–149)	147 (146–148)	140–155	**0.011**	0.055
K [mmol/L]*^,$^	4.5 (4.4–4.7)	4.6 (4.4–4.8)	3.5–5.1	0.458	1
Mg [mmol/L]^&^	0.9 (0.8–0.9)	0.9 (0.8–0.9)	0.6–1.3	0.310	1
***Trace elements***					
Fe [μmol/L]^$^	26 (23–31)	29 (24–34)	15–45	0.065	0.325
Cu [μmol/L]^#,&^	8.0 (7.2–8.9)	9.1 (8.1–9.9)	4.4–25.5	**0.001**	**0.005**
Zn [μmol/L]^#,$^	9.9 (8.5–11.1)	12.3 (11.1–14.0)	6.3–26.4	** < 0.001**	** < 0.001**
Mn [nmol/L]^#,&^	63 (55–71)	71 (64–79)	< 360	**0.007**	**0.035**
I [μmol/L]^#,&^	0.54 (0.37–1.06)	0.99 (0.80–1.30)	0.30–1.53	** < 0.001**	**0.001**
***Vitamins***					
Vit. D [nmol/L]^&^	151 (118–184)	156 (128–188)	48–350	0.879	1
Vit. E [μmol/L]^&^	33 (22–45)	71 (55–86)	7–56	** < 0.001**	** < 0.001**
Vit. A [μmol/L]^&^	3.2 (2.6–4.3)	3.2 (2.8–3.9)	2.9–10.7	0.810	1
Vit. B_12_ (cobalamin) [pmol/L]^&^	311 (267–384)	413 (331–497)	221–590	** < 0.001**	**0.001**
Vit. B_9_ (folate) [nmol/L]^&^	11 (8–16)	20 (15–26)	7–23	** < 0.001**	** < 0.001**

Summary statistics for the values (calculated as the means for both time points, TP1 and TP2, in each feeding group of dogs) obtained from mineral and vitamin analyses are reported as medians and interquartile ranges. RMBD = raw meat-based diet; CD = commercial diet; RI = reference interval; n/a = not available. ^§^*P*-values refer to the comparison of the average values calculated from both TP1 and TP2 data. *P*_*corr*_: Bonferroni correction of the significance level for each main category: *P*_*corr*_ < 0.01 (k = 5). ^&^Non-parametric comparison using the Mann–Whitney U test. ^$^Parametric comparison using an unpaired t test. *One dog with an unexplainable high value (7.6 mmol/L) was excluded from statistical analyses. ^#^Values obtained for 101 dogs (RMBD: n = 50, CD: n = 51) at TP1 and 101 dogs (RMBD: n = 48, CD: n = 53) at TP2

RMBD-fed dogs had significantly lower serum I, Cu, Zn, and Mn concentrations than CD-fed dogs (I: ES = 0.36, Cu: ES = 0.33, Zn: ES = 0.56, and Mn: ES = 0.27; Table 2); but serum Cu, Zn, and Mn concentrations remained within the RI in all dogs. Serum I concentrations exceeded the RI with a range from 1.55–1.94 μmol/L in 6 RMBD-fed dogs (12%) and with a range from 1.55–1.70 μmol/L in 4 CD-fed dogs (8%; calculated means for both time points, TP1 and TP2). Significantly lower serum concentrations were also detected for vitamin E in RMBD- *vs.* CD-fed dogs (ES = 0.74), showing serum vitamin E concentrations within the RI in 45 dogs (88%) and exceeding the RI with a range from 57–76 μmol/L in 6 RMBD-fed dogs (12%) at TP1. In contrast, 71% (n = 41) of CD-fed dogs had serum vitamin E concentrations exceeding the RI with values ranging from 57–127 μmol/L at TP1 (Add. file 5). Serum cobalamin concentrations were also significantly lower in RMBD-fed than in CD-fed dogs (ES = 0.37), but all dogs in both feeding groups had serum cobalamin concentrations within the RI.

## Discussion

Overweight and obesity are common conditions in dog populations in western countries [13, 27] with increasing prevalence [19]. In fact, over the past five decades, obesity has become the most common nutritional disease in dogs [12]. Management of diet and exercise was identified as one of the three major factors contributing to obesity in dogs [30]. An association between obesity and the number of meals fed to the dog [30] and the dog´s presence at the time of the owner consuming their meals has been reported [31]. Beyond the frequency of meals, studies on the body condition of dogs do not differentiate the type of diet that was fed to the animals. In the present study, RMBD-fed dogs were less frequently affected by overweight or obesity based on the assessment of BCS. In accordance with our study, Hiney et al. reported a significantly lower BCS in RMBD-fed dogs [10]. However, RMBD-fed dogs in this study were about 2.4 years older than the control group, which does not apply to RMBD-fed dogs of the present study, and CD-fed dogs were similar in age (5.1 years) [10] when compared to the present study (4.8 years). Applying our studies’ inclusion and exclusion criteria yielded a fairly homogeneous study population as described by the sex, age, body weight, and breeds of the dogs, eliminating the possibility of differences in demographics to present confounding factors on body constitution data and nutritional status. Statistical analyses confirmed this homogeneity of the study population. However, a limitation of this study was that the data obtained regarding feed intake (e.g., type and amounts of daily feedstuffs) and physical activity data depended on the accuracy of the information provided by the dog owners. Furthermore, in RMBD rations, information on nutrient intake relied on tabled values of the various feedstuffs. In contrast, labelled nutrients provided by the manufacturer, based on measured values, were used for ration calculation in CD-fed dogs. RMBD-fed dogs were provided an average of 89% of the daily NRC recommendation for ME, calculated based on the body weight of the dog. It can be reasonably concluded that this lower energy intake resulted in a significantly lower BCS than those in CD-fed dogs (1 score point difference). A possible factor contributing to a lower ME intake for RMBD-fed dogs could be a lower energy density in RMBDs than in CDs for dogs [32]. RMBDs are, therefore, fed in larger quantities per meal without exceeding the daily recommended ME intake. In contrast, one other study found a significantly higher energy content in RMBDs compared to CDs [33]. In the present study, RMBD rations provided approximately 54% of energy via protein versus 27% energy via protein in CD rations. Diets high in protein and low in carbohydrates are commonly recommended to achieve intentional body weight loss [34, 35]. The high content of protein preserves fat-free body mass. Carbohydrates are a more efficient source of energy, and diets high in carbohydrates result in a faster increase in blood glucose levels than diets high in protein [36]. A low amount of carbohydrates can result in weight loss or prevent weight gain [37]. CD-fed dogs in the present study were slightly overweight and were provided 102% of the daily NRC recommendation for ME, while 46% of the energy was provided by carbohydrates as the main source of energy supply in CD rations. On the other hand, RMBD rations contained a higher amount of fat than CD rations. High carbohydrate and high-fat feeding may contribute to insulin resistance by reducing β-cell sensitivity in overweight dogs [38, 39].

In our study, dog owners were asked to assess the BCS of their dog. Interestingly, the owners' assessment of their dogs´ BCS was consistent with the veterinarian’s assessment in RMBD-fed dogs but not in CD-fed dogs. This discrepancy is similar to findings in other studies reporting a relatively low percentage (52–65%) of dog owners – regardless of the feeding regime – being able to correctly estimate their dog´s body condition [19, 40, 41]. A possible explanation is that owners who elect an RMBD are generally more involved in the topic of nutrition and, therefore, presumably better informed about the health effects of diets and correct assessment of the body condition of their dog. In the present study, owners were not shown a BCS scale prior to assessment. However, a previous study showed that correct BCS assessment for a dog by the owners does not depend on the availability of BCS charts [41]. It was reported that owners of sporting dogs assessed their dogs´ BCS more accurately than pet dog owners [40]. These findings align with data of the present study, as more RMBD-fed dogs were regularly engaged in dog sporting activities. Studies showed that dog owners and veterinarians tend to agree in body constitution assessment when dogs are of normal weight or underweight, but estimations are more likely to differ when dogs are overweight [42, 43]. CD-fed dogs of the present study were slightly overweight and owners of CD-fed dogs tended to estimate the BCS of their dog lower than the veterinarian. The fact that owner assessment correlated positively with the veterinarian’s assessment supports the conclusion that owners of CD-fed dogs did not arbitrarily misjudge their dogs´ body condition but that the assessment overall was shifted to a lower rank. NRC provides different recommendations on ME intake for dogs under maintenance conditions as compared to those that are normally active [44]. In the present study, all dogs were assumed to have an active lifestyle. According to our results, a 90% coverage of the NRC recommendation for ME was sufficient to maintain an ideal range of BCS with an active lifestyle. However, although energy intake was below the estimated requirement, RMBD-fed dogs were not underweight. This result aligns with the fact that most studies determining energy requirements for adult dogs were conducted on kennel dogs [44] under maintenance conditions, which might not be accurate for most pet dogs with regular exercise [45]. RMBD-fed dogs were more active and participated more frequently in dog sports. Data on the activity of the dogs were based on owner-provided information via a standard study questionnaire. Interestingly, however, statistical analyses found that the time spent actively per week was not related to the BCS of the dogs in both feeding groups. However, more RMBD-fed dogs engaged in regular dog sporting activities than CD-fed dogs, and dog sports presented a higher intensity of activity during the active period. These results are in accordance with the results of others showing dogs participating in dog sporting activities to have a lower BCS than pet dogs that do not engage in dog sporting activities [40].

In the present study, we calculated coverage of the recommendation for Ca, P, and Na at around 100% in RMBD rations, whereas the recommended daily intakes were exceeded by over 200% for Ca, P, and Na in the CD rations. For most RMBD rations, a Ca-containing supplement was used which explains the well-balanced intake of the above-mentioned minerals. Our findings do not match with the results of other studies over the past decade, which indicate RMBD rations are deficient in Ca [11, 46]. However, our results agree with a recent study, where 69% of the RMBD rations met the NRC recommendation for Ca [33]. It could be speculated that pet owners today are more aware of potential mineral deficiencies in RMBD rations than in the past. Despite Ca and P supply being close to 100% of the NRC recommendation, the Ca:P ratio was relatively narrow at only 1:1 in RMBD rations (CD: 1.2:1). The recommendation for Ca and P intake should provide a ratio between the two minerals of 1.2:1 [44] or between 1:1 and up to 2:1, with an optimum of 1.4:1 [47]. Results of our study align with other reports of inverse Ca:P ratios in RMBD rations [11, 33]. The Ca:P ratio, in turn, can affect the absorption of Ca and P [48] as high P and low Ca levels in the diets enhance intestinal Ca absorption in dogs and may induce nutritional secondary hyperparathyroidism [49]. While adult dogs seem to tolerate an inverse Ca:P ratio well, this may lead to developmental orthopedic conditions in growing dogs [50]. RMBD-fed dogs did not show any clinical signs of disorders in bone metabolism. Ca and P serum concentrations were within the recommended RI for both diets; however, it was shown that calculated imbalances of diets do not correlate with blood parameters [33]. Furthermore, clinical signs of Ca deficiency may occur as late as 18–24 months after a diet change to an RMBD based on empirical observations [51]. Dogs in the present study were fed an RMBD for at least 12 months prior to the first presentation, and possible long-term consequences of permanent Ca deficiency were not within the scope of our study.

A lack of calculated Cu, Zn, Mn, and I intake was detected in RMBD rations, which is consistent with other studies [11, 33, 46, 52, 53]. In line with the lower intake of Cu, Zn, and Mn in RMBD-fed dogs, the serum concentrations of Cu, Zn, and Mn were also significantly lower than in CD-fed dogs. This was previously reported for Cu and Zn serum concentrations in RMBD-fed dogs [33].

Mn, as an important co-factor for various enzymes in mammals [44], is absorbed in the small intestine [54]. While sufficient Mn intake is linked to fertility in female dogs and essential for embryonic survival [55], there are no reports of Mn deficiency and its health consequences in dogs. In the present study, RMBD rations covered approximately 18% of the daily recommended intake for Mn, but Mn serum concentrations were lower in RMBD-fed dogs. This is contrary to findings in another study, where RMBD rations also did not cover the daily recommendation for Mn. However, Mn serum concentrations did not differ between CD- and RMBD-fed dogs [56]. Another study on RMBDs found a 91% median coverage of the recommendation for Mn [11].

The present study confirms previously reported differences in the estimated NRC recommendation coverage for I, which was lower in RMBD rations than in CD rations [11, 33]. Foodstuffs rich in I include seaweed and fish, and a lack of I in rations that do not contain these foodstuffs is likely without I supplementation [11, 57]. Only about half of the RMBD rations of the present study contained fish, seaweed, or an I supplement, and an I intake below recommendation seemed likely. As I is critical for thyroid hormone production, clinical signs of I deficiency are those of hypothyroidism, including lethargy, weight gain, and alterations of skin and coat such as alopecia, dry or seborrheic skin, and hyperpigmentation [58, 59]. Deficiencies of I during pregnancy and lactation can also lead to dwarfism or delayed growth (congenital hypothyroidism) in puppies [60]. Despite a calculated deficit of I supply in RMBD-fed dogs, these dogs did not show clinical signs of hypothyroidism or goiter. It remains to be clarified whether the I deficiency was subclinical, as the dogs received the RMBD for one year prior to inclusion in the study. Severe clinical signs of hypothyroidism have been reported in dogs after being fed home-cooked diets lacking I for 12 months [61]. In the present study, serum total T4 and TSH activities were within the RI in all dogs (reference to paper 2), suggesting sufficient I intake. Alternatively, RMBD-fed dogs may have adapted to a low I intake as shown by others [62].

We acknowledge that this study was not without limitations. Key variables, including dietary intake and physical activity, were based on owner self-report and therefore a small risk of recall and potential social desirability bias cannot be excluded. However, the use of owner-reported data was intentional, as the primary aim of the present study was to assess feeding practices under field conditions rather than controlled experimental settings. Physical activity and body condition were evaluated using approaches appropriate for a heterogeneous, field study population. Although objective measurements such as accelerometry could provide additional insight, their utilization in owner-managed settings presents practical challenges. Future studies are expected to build on the present findings by integrating objective activity measures where possible.

## Conclusion

Obesity is the most common dietary disorder in dogs. As the management of diet and exercise emerges as a crucial factor contributing to canine obesity, RMBD diets could be an approach to prevent development of an overweight status. In the present study, a lower BCS observed in RMBD-fed dogs was attributed mostly to dietary factors such as lower energy intake and higher protein content in RMBDs, which may help in weight management by preserving fat-free body mass and reducing fat accumulation. Owner assessment of their dogs´ body condition in CD-fed dogs ranked lower but paralleled the veterinarian’s assessment, suggesting that with improved client education by veterinary staff, BCS assessment by the owners can be optimized to further sensitize for overweight or obesity in dogs. Several nutritional imbalances were revealed in RMBD diets. Dietary assessment and correction of RMBD rations by a veterinarian or veterinary nutritionist, as well as the use of supplements, where necessary, are therefore recommended to ensure a healthy balanced diet. This study underlines the importance of tailored nutritional management in canine obesity prevention and highlights the need for further research to elucidate the complex interactions between diet, activity levels, and health outcomes in dogs.

## Material and Methods

### Ethics approval

The study was reviewed and approved by the Thuringia State Office for Consumer Protection (*Landesamt für Verbraucherschutz Thüringen, Germany; registration no. UNL-22–001*). The owners of each dog enrolled in the study gave their written informed consent to participate in the study. In addition, the dog’s owner signed a personal data protection statement form (EU General Data Protection Regulation).

### Animals and study inclusion criteria

Recruitment of dogs for the study was via an information flyer that was displayed in 4 veterinary practices in the city of Erfurt, Thuringia, Germany. In addition, some dog owners received information about the study online (*Facebook*) through relevant groups (i.e., a group of owners feeding RMBD and a group of dog owners from the respective geographical area). Owners were asked to initiate enrollment of their dog in the study by sending a written response with basic information about the age, breed, and type of feeding for their dog to one of the authors (LVL). Criteria to exclude dogs from the study were: (i) age < 2 years or > 8 years (i.e., only dogs in the adult phase were included), (ii) body weight < 6 kg or > 35 kg (given the amount a sampling material required for the study), and having a medical condition or receiving long-term medication (other than preventative treatment).

As an inclusion criterion for the study, each dog had to be fed the current dietary plan exclusively for at least 12 months. Based on that dietary history, the dogs were dichotomously assigned to either the RMBD group (= observation group fed a raw meat-based diet) or the CD group (= control group fed a dry and/or canned commercial non-therapeutic complete diet).

All dogs (n = 113) considered for enrollment in the study were presented at a veterinary clinic (*Erfurt, Germany*) between September 2022 and April 2023 for further diagnostic evaluation.

Here, dogs of both feeding groups, were evaluated twice by a veterinarian (LVL) at intervals of 3–5 months to exclude short-term effects of feeding and/or changes in the dog’s overall health status (Fig. 11). Most dogs were initially presented during the fall and winter months (September – December; time point [TP] 1) and were re-evaluated in spring of the following year (February – April, TP2). In between TP1 and TP2, the dogs were housed in their usual home environments with their owners and resumed their normal activities and feeding schedules. Nine of the enrolled dogs were excluded from the analysis due to (i) missing TP2 (CD: n = 2; RMBD: n = 1), (ii) not fulfilling the inclusion criteria for body weight (CD: n = 1; RMBD: n = 1), (iii) long-term corticosteroid administration (CD: n = 1), or (iv) a diagnosis of hypothyroidism (RMBD: n = 2) or diabetes mellitus (CD: n = 1). Thus, a total of 104 healthy dogs were included in the final data analysis.

In addition to a thorough physical examination, the body weight of the dogs was documented at each TP using a digital scale (*Soehnle Professional 7858*). A possible difference in body weight (${\Delta }_{bw})$ between the two TP was calculated as ${\Delta }_{bw}[\%]=\frac{\left(b{w}_{2}\left[kg\right]-b{w}_{1}[kg]\right)}{b{w}_{1}[kg]}$ (where $b{w}_{2}$ = body weight of the dog at TP2, and $b{w}_{1}$ = body weight of the dog at TP1). Each dog was further assessed by estimating the BCS on a scale from 1 to 9 [63, 64]and the MCS according to the standardized scheme of the WSAVA Global Nutrition Committee [65]. Both BCS and MCS of the dogs were determined at each visit and by the same investigator (LvL). Documentation of the nutritional status also included photographs of the dogs available for review and later re-evaluation.

A standardized study questionnaire served to record the dog’s signalment (age, sex, reproductive status, breed) and relevant medical history (annual vaccination protocol and deworming schedule) to determine the dog’s overall health and obtain a semi-quantitative assessment of the average daily physical activity of the dog (documented as the time spent with physical activity in hours per 24 h on weekdays and weekends and whether the dogs participated in dog sports). The mean time spent on physical activity in hours per week was calculated for each dog. For dogs participating in dog sports, the cumulative active time was calculated by adding the time spent on sporting activities in hours per week to the mean time spent on physical activity per week.

### Dietary history and ration calculation

A complete dietary history was obtained for each dog by use of a standard study questionnaire (Add. file 3 + 4). For CD-fed dogs, information was required about the manufacturer, the complete name of the commercial food, and the exact amount of food that was fed daily. In addition, any treats fed to the dog (e.g., carrots, cheese) had to be recorded; for relevant amounts of treats, these were included in the ration calculation. Whenever possible, owners provided information about the original composition and/or analytical components of the CD or the ingredients of the RMBD (e.g., package label).

For RMBD-fed dogs, a detailed list of all ingredients and amounts fed daily was provided by the owners. This included the type (or types) of meat or boned meat, fruit, and vegetable(s), the carbohydrate source (if used), and additives for mineral and trace element supplementation. In addition, the acquisition of the raw meat ingredient was documented (e.g., purchased as fresh meat at the supermarket, butcher shop, or special BARF store or obtained as frozen meat). Motivations for dog owners to elect feeding an RMBD to their dog were recorded in a free text format, where multiple aspects were grouped based on context. The composition of each ration was verified with the owners at TP2, and changes in the ration were documented (if applicable). All dog owners were educated on the necessity of correct ration information.

Calculation of the nutritional composition of the rations was performed for each diet using a commercial ration formulation calculator (*napfcheck*^®^*, Dr. med. vet. Julia Fritz, Planegg, Germany; available at:* https://www.napfcheck.de/). Whenever possible, labelled nutrients and analytical components of the respective ingredients were added to the calculation. If no labelling was provided by the owners, the table-listed values available through the ration formulation program were used as the basis to calculate the ration. Calculation of the ration contents was based on the amount of each ration component fed according to the owners. For each dog, the nutrient and energy intakes were compared with the respective recommendations [44].

### Specimen collection and analysis

An aliquot (spot sample) of naturally-passed feces and a free-catch urine sample were obtained from each dog at both study visits and were used to perform routine in-house fecal parasitology (direct smear and flotation analysis) and urinalysis (urine dipstick, microscopic urine sediment analysis, and refractometric determination of urine specific gravity). Whole blood samples were used for in-house hematology analysis, and serum samples were obtained for further clinicopathologic testing (for details, see companion article: reference to paper 2). Serum Ca, P, Mg, and Fe concentrations were photometrically analyzed (*Cobas*^®^* 8000 modular analyzer; Roche; Switzerland*), and serum vitamin B_12_ (cobalamin) and vitamin B_9_ (folate) concentrations were measured using chemiluminescence assays (LIA; *Cobas*^®^* 8000 modular analyzer; Roche*). Analysis of serum Na and K concentrations was done by potentiometry (POT) (*Cobas*^®^* 8000 modular analyzer; Roche*). Serum trace element concentrations were determined by inductively coupled plasma–mass spectrometry (ICP-MS; *ICP MS-2023; Shimadzu; Japan*). Analysis of serum vitamin D concentration was performed by LIA (*ADVIA Centaur XPT; Siemens; Germany*) and of vitamin E and vitamin A concentrations by high-performance liquid chromatography (HPLC; *LC-20; Shimadzu*). Interpretation of the results was based on the reference intervals (RI) established and reported by the diagnostic laboratory where the analyses were performed (*Laboklin, Bad Kissingen, Germany)*.

### Statistical analysis

Commercially available software (*SPSS v.20; IBM, Chicago, IL, USA*) was used for all statistical analyses, and Excel (*Microsoft Office 2021, Redmond, WA, USA*) was used to generate graphical representations of the data. The level of statistical significance was set at *P* < 0.05 for all 2‐sided analyses and – if indicated – was corrected for each category of results via Bonferroni calculation accounting for the number of correlation analyses (k) performed at the same level. Normality of the data was assessed visually and quantitatively using a Shapiro‐Wilk test. Depending on the data distribution, an independent *t*‐test (parametric data) or Mann‐Whitney *U*‐test (nonparametric data) was used for comparisons between the two feeding groups of dogs (RMBD *vs.* CD). Paired data analyses were performed using a Wilcoxon signed‐rank test (e.g., assessment of the dog’s BCS by the veterinarian *vs*. the owner). Continuous variables obtained at both visits (e.g., nutritional ration composition) were tested for homogeneity within each dog by using a Wilcoxon signed‐rank test (level of statistical significance corrected to *P* < 0.003 for multiple analyses at the same level, including minerals and vitamins). Based on consistent homogeneity of the estimated nutritional ration composition and serum mineral and vitamin data, the average values (arithmetic means) of the results obtained at the two evaluations (TP1 and TP2) were used for statistical analyses. Numerical outliers were identified by calculating the corresponding outer Tukey fences (defined as 3 times the interquartile range (IQR) above the 75th percentile or below the 25th percentile of the data). Treatment of outliers was based on the consideration as either “inside the calculated outer Tukey fence” (ITF; mild outliers) or “outside the calculated outer Tukey fence” (OTF; severe outliers). Categorical variables (e.g., breed, sex, vaccination status) were compared between the two feeding groups of dogs by using a chi-square test (when expected cell values were > 5) or Fisher's exact tests (with expected cell values ≤ 5). ES was calculated as Cohen´s *d* for normally distributed data and as Pearson's *r* correlation coefficient for non-normally distributed data. ES < 0.3 was interpreted as weak, 0.3 ≤ ES < 0.5 as medium, and ES > 0.5 as a strong difference or relationship, according to Cohen [66]. A Spearman correlation coefficient *ρ* was calculated to assess the direction and strength of possible relationships between continuous and/or ordinal data (e.g., time spent on activity with the dogs, time spent on dog sporting activities, mean coverage of recommended ME, and BCS of the dogs). Interpretation of the Spearman coefficient was based on Cohen's limits, with *ρ* < 0.10 indicating a weak correlation, 0.10 > *ρ* < 0.30 a moderate correlation, and *ρ* > 0.50 a strong correlation [67].

## Supplementary Information

Additional file 1. Supplementary information on the breeds of n=104 healthy dogs included in the study.

Additional file 2. Change in body weight (in %) of n=104 dogs between the 1 st and 2nd time point (TP). The change in body weight was numerically higher in CD-fed dogs (median: 1.3%, interquartile range [IQR]: -0.4–3.3%) than in RMBD-fed dogs (median: 0%, IQR: -3.4–1.8%), but the difference was not significant (P=0.938). The length of the box represents the IQR, the horizontal line of the box is the median, and the whiskers represent 1.5-times the IQR below the 25th quartile and above the 75th quartile; the dot represents an outlier (ITF); the star represents an extreme outlier (OTF). The dashed line marks a 0% change in body weight. RMBD = raw meat-based diet; CD = commercial diet.

Additional file 3. Scatter plot representing the correlation of BCS and the mean time spent on activity (in hours/week [h/wk]) for n=104 dogs fed either an RMBD or CD. Each dot represents an individual. No strong correlation of the two shown parameters could be found. Line of best fit (Correl.) calculation for CD: Polynomial regression: y = -0.0086x2 + 0.1904x + 4.6618; R² = 0.0141. Line of best fit (Correl.) calculation for RMBD: Polynomial regression: y = -0.0087x2 + 0.2086x + 3.5954; R² = 0.0341. RMBD = raw meat-based diet; CD = commercial diet.

Additional file 4. Scatter plot representing the correlation of BCS and the coverage of the daily recommendation for metabolizable energy (ME; in %) for n=104 dogs fed either an RMBD or CD. Each dot represents an individual. Spearman correlation analysis revealed a weak negative correlation between the coverage of recommendation for ME and BCS of the dogs (P=0.011; effect size [ES]=0.25). Line of best fit (Correl.) for CD: Polynomial regression: y = 0.0002x2–0.0582x + 9.4011; R² = 0.134. Line of best fit (Correl.) for RMBD: Polynomial regression: y = 6E-05x2 - 0.0266x + 6.5874; R² = 0.1787. RMBD = raw meat-based diet; CD = commercial diet.

Additional file 5. Results of mineral and vitamin analysis in blood serum samples from n=104 healthy dogs included in the study.

Additional file 6. Study questionnaire for RMBD-feeding dog owners used in the study.

Additional file 7. Study questionnaire for CD-feeding dog owners used in the study.

## Data Availability

The dataset used and analyzed for the current study are available from the corresponding author upon reasonable request.

## Abbreviations

BARF: Biologically appropriate raw food
BCS: Body condition score
Ca: Calcium
CBC: Complete blood cell count
CD: Commercial diet
CF: Crude fiber
Cu: Copper
ES: Effect size
DM: Dry matter
Fe: Iron
I: Iodine
ICP-MS: Inductively coupled plasma–mass spectrometry
ITF: Inside the calculated outer Tukey fence
IQR: Interquartile range
HPLC: High-performance liquid chromatography
K: Kalium
LIA: Chemiluminescence immunoassay
ME: Metabolizable energy
MCS: Muscle condition score
Mg: Magnesium
Mn: Manganese
Na: Natrium
OTF: Outside the calculated outer Tukey fence
P: Phosphorous
POT: Potentiometry
RI: Reference interval
RMBD: Raw meat-based diet
TP: Time point
Zn: Zinc
