# Supplementary material for: Lower prevalence of obesity and nutritional imbalances in dogs fed a raw meat-based diet (RMBD) compared to a commercial complete diet
Source: BMC Vet Res. 2026 Feb 6;22:127. doi: 10.1186/s12917-026-05283-4 (PMC12930774; doi:10.1186/s12917-026-05283-4)
Supplement: Supplementary file 1 — Additional file 1. Supplementary information on the breeds of n=104 healthy dogs included in the study. [file 12917_2026_5283_MOESM1_ESM.pdf]

**Additional file 1.** Supplementary information on the breeds of n=104 healthy dogs included in the study.

| **Breed** | **RMB-fed dogs, n** | **CD-fed dogs, n** |
|---|---|---|
| Akita Inu | - | 1 |
| Australian Cattle Dog | 1 | 1 |
| Australian Shepherd | 1 | 1 |
| Miniature Australian Shepherd | 1 | - |
| Bavarian Mountain Hound | 1 | - |
| Beagle | 1 | 1 |
| Bearded Collie | - | 1 |
| Beauceron | - | 1 |
| Bobtail | 1 | - |
| Border Collie | 2 | 3 |
| Dachshund | 2 | - |
| Miniature Dachshund | - | 1 |
| Dalmatiner | 1 | - |
| Dobermann | 1 | - |
| Dogo Canario | - | 1 |
| Duck Tolling Retriever | 1 | - |
| French Bulldog | 3 | 1 |
| Retro French Bulldog | - | 1 |
| German Boxer | 1 | 1 |
| German Shepherd | 2 | - |
| Giant Poodle | - | 1 |
| Giant Schnauzer | 2 | - |
| Golden Doodle | 3 | 2 |
| Golden Retriever | 1 | 2 |
| Harzer Fuchs | 1 | - |
| Hovawart | - | 2 |
| Labrador Retriever | 2 | 7 |
| Lagotto Romagnolo | 2 | - |
| Magyar Vizsla | 1 | - |
| Malinois | - | 2 |
| Olde English Bulldog | 2 | - |
| PON (Polski Owczarek Nizinny) | - | 1 |
| Retro Pug | 1 | 1 |
| Rhodesian Ridgeback | - | 1 |
| Shiba Inu | - | 1 |
| Siberian Husky | 2 | - |
| Staffordshire Bullterrier | 2 | 1 |
| Weimaraner | 1 | - |
| Wolfhound | 2 | - |
| The different breeds of purebred dogs included in the study are listed in alphabetical order and the number of dogs of each breed is summarized as counts (n). | | |
