# Supplementary material for: Lower prevalence of obesity and nutritional imbalances in dogs fed a raw meat-based diet (RMBD) compared to a commercial complete diet
Source: BMC Vet Res. 2026 Feb 6;22:127. doi: 10.1186/s12917-026-05283-4 (PMC12930774; doi:10.1186/s12917-026-05283-4)
Supplement: Supplementary file 2 — Additional file 2. Change in body weight (in %) of n=104 dogs between the 1 st and 2nd time point (TP). The change in body weight was numerically higher in CD-fed dogs (median: 1.3%, interquartile range [IQR]: -0.4–3.3%) than in RMBD-fed dogs (median: 0%, IQR: -3.4–1.8%), but the difference was not significant (P=0.938). The length of the box represents the IQR, the horizontal line of the box is the median, and the whiskers represent 1.5-times the IQR below the 25th quartile and above the 75th quartile; the dot represents an outlier (ITF); the star represents an extreme outlier (OTF). The dashed line marks a 0% change in body weight. RMBD = raw meat-based diet; CD = commercial diet. [file 12917_2026_5283_MOESM2_ESM.pdf]

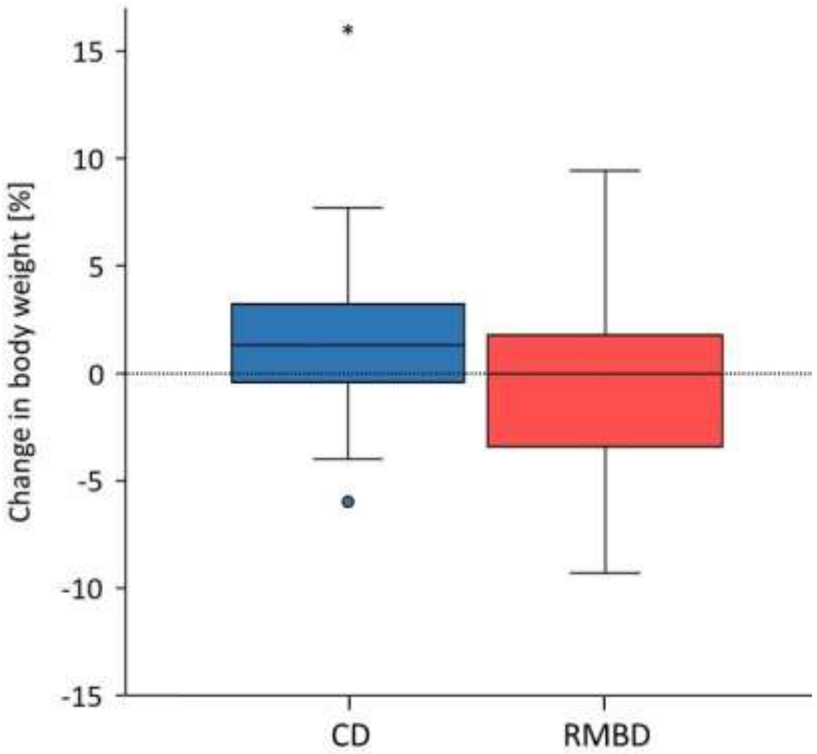

Additional file 2. Change in body weight (in %) of n=104 dogs between the 1st and 2nd time point (TP). The change in body weight was numerically higher in CD-fed dogs (median: 1.3%, interquartile range [IQR]: -0.4–3.3%) than in RMBD-fed dogs (median: 0%, IQR: -3.4–1.8%), but the difference was not significant (*P*=0.938). The length of the box represents the IQR, the horizontal line of the box is the median, and the whiskers represent 1.5-times the IQR below the 25th quartile and above the 75th quartile; the dot represents an outlier (ITF); the star represents an extreme outlier (OTF). The dashed line marks a 0% change in body weight. RMBD = raw meat-based diet; CD = commercial diet.
