# Supplementary material for: Lower prevalence of obesity and nutritional imbalances in dogs fed a raw meat-based diet (RMBD) compared to a commercial complete diet
Source: BMC Vet Res. 2026 Feb 6;22:127. doi: 10.1186/s12917-026-05283-4 (PMC12930774; doi:10.1186/s12917-026-05283-4)
Supplement: Supplementary file 3 — Additional file 3. Scatter plot representing the correlation of BCS and the mean time spent on activity (in hours/week [h/wk]) for n=104 dogs fed either an RMBD or CD. Each dot represents an individual. No strong correlation of the two shown parameters could be found. Line of best fit (Correl.) calculation for CD: Polynomial regression: y = -0.0086x2 + 0.1904x + 4.6618; R² = 0.0141. Line of best fit (Correl.) calculation for RMBD: Polynomial regression: y = -0.0087x2 + 0.2086x + 3.5954; R² = 0.0341. RMBD = raw meat-based diet; CD = commercial diet. [file 12917_2026_5283_MOESM3_ESM.pdf]

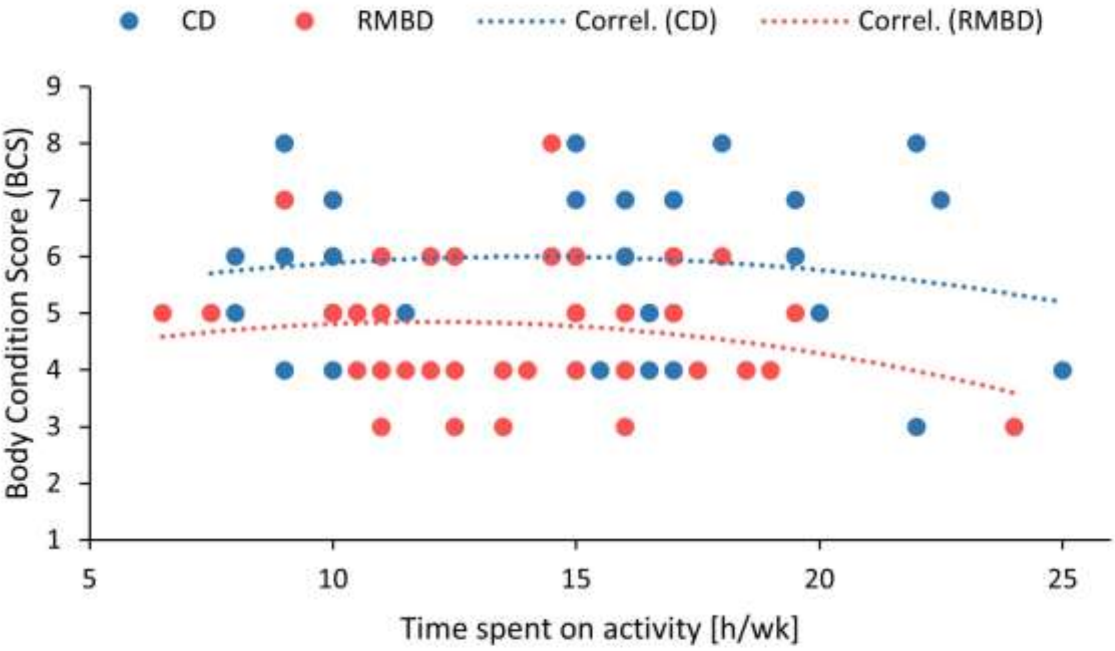

Additional file 3. Scatter plot representing the correlation of BCS and the mean time spent on activity (in hours/week [h/wk]) for n=104 dogs fed either an RMBD or CD. Each dot represents an individual. No strong correlation of the two shown parameters could be found. Line of best fit (Correl.) calculation for CD: Polynomial regression: $y = -0.0086x^2 + 0.1904x + 4.6618$; $R^2 = 0.0141$. Line of best fit (Correl.) calculation for RMBD: Polynomial regression: $y = -0.0087x^2 + 0.2086x + 3.5954$; $R^2 = 0.0341$. RMBD = raw meat-based diet; CD = commercial diet.
