# Supplementary material for: Lower prevalence of obesity and nutritional imbalances in dogs fed a raw meat-based diet (RMBD) compared to a commercial complete diet
Source: BMC Vet Res. 2026 Feb 6;22:127. doi: 10.1186/s12917-026-05283-4 (PMC12930774; doi:10.1186/s12917-026-05283-4)
Supplement: Supplementary file 4 — Additional file 4. Scatter plot representing the correlation of BCS and the coverage of the daily recommendation for metabolizable energy (ME; in %) for n=104 dogs fed either an RMBD or CD. Each dot represents an individual. Spearman correlation analysis revealed a weak negative correlation between the coverage of recommendation for ME and BCS of the dogs (P=0.011; effect size [ES]=0.25). Line of best fit (Correl.) for CD: Polynomial regression: y = 0.0002x2–0.0582x + 9.4011; R² = 0.134. Line of best fit (Correl.) for RMBD: Polynomial regression: y = 6E-05x2 - 0.0266x + 6.5874; R² = 0.1787. RMBD = raw meat-based diet; CD = commercial diet. [file 12917_2026_5283_MOESM4_ESM.pdf]

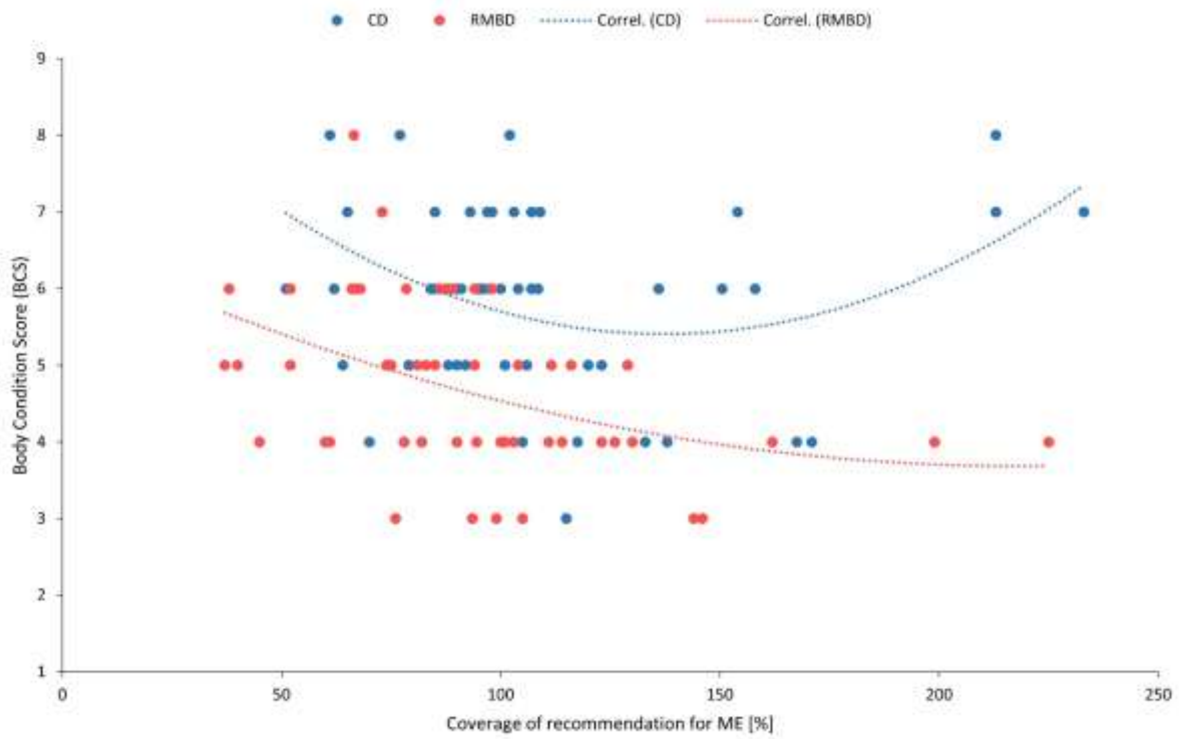

Additional file 4. Scatter plot representing the correlation of BCS and the coverage of the daily recommendation for metabolizable energy (ME; in %) for n=104 dogs fed either an RMBD or CD. Each dot represents an individual. Spearman correlation analysis revealed a weak negative correlation between the coverage of recommendation for ME and BCS of the dogs (*P*=0.011; effect size [ES]=0.25). Line of best fit (Correl.) for CD: Polynomial regression: $y = 0.0002x^2 - 0.0582x + 9.4011$; $R^2 = 0.134$. Line of best fit (Correl.) for RMBD: Polynomial regression: $y = 6E\text{-}05x^2 - 0.0266x + 6.5874$; $R^2 = 0.1787$. RMBD = raw meat-based diet; CD = commercial diet.
