# Supplementary material for: Lower prevalence of obesity and nutritional imbalances in dogs fed a raw meat-based diet (RMBD) compared to a commercial complete diet
Source: BMC Vet Res. 2026 Feb 6;22:127. doi: 10.1186/s12917-026-05283-4 (PMC12930774; doi:10.1186/s12917-026-05283-4)
Supplement: Supplementary file 5 — Additional file 5. Results of mineral and vitamin analysis in blood serum samples from n=104 healthy dogs included in the study. [file 12917_2026_5283_MOESM5_ESM.pdf]

Additional file 5. Results of mineral and vitamin analysis in blood serum in n=104 healthy dogs included in the study.

Parameter	RMBD-fed dogs, TP1	RMBD-fed dogs, TP2	CD-fed dogs, TP1	CD-fed dogs, TP2	RI	$P^{§}$	P_{corr}
n	51		53				
Major minerals[&]							
Total Ca [mmol/L]	2.5 (2.4-2.6)	2.5 (2.4-2.6)	2.5 (2.5-2.6)	2.5 (2.5-2.6)	2.3-3.0	**0.006**	**0.030**
P [mmol/L]	1.2 (1.1-1.3)	1.2 (1.1-1.3)	1.2 (1.0-1.3)	1.2 (1.1-1.3)	0.7-1.6	0.420	1
Na [mmol/L]	147 (146-149)	148 (147-148)	147 (146-148)	147 (146-148)	140-155	0.011	0.055
K [mmol/L]*	4.5 (4.4-4.7)	4.5 (4.4-4.7)	4.6 (4.4-4.8)	4.6 (4.4-4.8)	3.5-5.1	0.458	1
Mg [mmol/L]	0.8 (0.8-0.9)	0.9 (0.8-0.9)	0.8 (0.8-0.9)	0.8 (0.8-0.9)	0.6-1.3	0.310	1
Trace elements[&]							
Fe [µmol/L]	28 (23-32)	25 (22-31)	28 (24-32)	27 (24-37)	15-45	0.065	0.325
Cu [µmol/L][#]	7.9 (7.2-8.7)	8.0 (7.1-9.0)	9.0 (7.7-9.8)	9.3 (8.2-10.1)	*n/a*	**0.001**	**0.005**
Zn [µmol/L][#]	9.8 (8.5-11.0)	9.8 (8.6-11.2)	11.9 (11.0-13.7)	12.7 (11.2-14.4)	*n/a*	**<0.001**	**<0.001**
Mn [nmol/L][#]	65.2 (56.6-73.8)	61.7 (55.6-67.3)	68.0 (59.7-74.3)	70.2 (64.1-80.6)	<360	**0.007**	**0.035**
I [µmol/L][#]	0.49 (0.34-1.04)	0.48 (0.33-0.87)	0.97 (0.70-1.26)	0.97 (0.77-1.27)	*n/a*	**<0.001**	**0.001**
Vitamins[&]							
Vit. D [nmol/L]	149 (118-187)	161 (115-197)	155 (122-193)	152 (126-182)	48-350	0.879	1
Vit. E [µmol/L]	33 (21-46)	33 (21-42)	67 (52-84)	72 (58-94)	7-56	**<0.001**	**<0.001**
Vit. E below RI, n (%)	-	1 (2)	1 (2)	-			
Vit. E above RI, n (%)	6 (12)	4 (8)	18 (34)	41 (77)			
Vit. A [µmol/L]	3.4 (2.7-4.2)	3.0 (2.6-4.2)	3.2 (2.7-4.4)	3.4 (3.0-4.2)	2.9-10.7	0.810	1
Cobalamin (vit. B_{12}) [pmol/L]	311 (258-373)	319 (286-388)	403 (337-513)	414 (326-478)	221-590	**<0.001**	**0.001**
Folic acid (vit. B_9) [nmol/L]	10 (6-14)	12 (9-16)	18 (13-28)	19 (14-29)	7-23	**<0.001**	**<0.001**
Vit. B_9 below RI, n (%)	16 (31)	-	4 (8)	2 (4)			
Vit. B_9 above RI, n (%)	5 (10)	-	17 (32)	21 (40)			

Results of mineral and vitamin analysis are summarized as medians and interquartile ranges (IQR) or counts (n) and proportions (%). RMBD = raw meat-based diet; CD = commercial diet; TP = time point; RI = reference interval; n/a = not applicable. [§]*P*-values refer to the comparison of the average values calculated from both TP1 and TP2. P_{corr}: Bonferroni correction of the significance level: [&]p_{corr}<0.010 (k=5). *One dog with an unplausible high value excluded from statistical analyses. [#]Values obtained for n=101 (RMBD: n=50; CD: n =51) dogs at TP1 and n=101 (RMBD: n=48; CD: n =53) dogs at TP2.
