# Supplementary material for: Lower prevalence of obesity and nutritional imbalances in dogs fed a raw meat-based diet (RMBD) compared to a commercial complete diet
Source: BMC Vet Res. 2026 Feb 6;22:127. doi: 10.1186/s12917-026-05283-4 (PMC12930774; doi:10.1186/s12917-026-05283-4)
Supplement: Supplementary file 6 — Additional file 6. Study questionnaire for RMBD-feeding dog owners used in the study. [file 12917_2026_5283_MOESM6_ESM.pdf]

Study questionnaire: Dogs receiving a raw meat-based diet (RMBD)

1. Patient information

- Name: ____
- Date of birth: ______________________________________
- Breed: ____
- Sex
  - ○ Male intact
  - ○ Male neutered
  - ○ Female intact
  - ○ Female spayed
- Does your dog have any known medical conditions?
  - ○ No
  - ○ Yes: ____
- Does your dog receive any long-term medication?
  - ○ No
  - ○ Yes: ____
- Does your dog live with partner animals in the same household, especially other dogs?
  - ○ No
  - ○ Yes: ____
  - If you checked "yes": What diet(s) do the other pets receive?
  - ○ Commercial complete food
    Please provide information about the manufacturer, complete name, and type of food (e.g., wet/canned or dry/kibble food): ____________________________________
  - ○ Raw meat-based diet (RMBD)
  - ○ Other: ____

2. Annual deworming schedule and vaccination protocol

- Deworming schedule – how often is your dog dewormed?
  - ○ 1x per year (every 12 months)
  - ○ 2x per year (every 6 months)
  - ○ 3x per year (every 4 months)
  - ○ 4x per year (every 3 months)
  - ○ Based on the results of routine fecal parasitology examination
  - ○ Other: ____
  - Dewormer (drug) used: ____
- Vaccination protocol – what is your dog vaccinated against?
  - ○ Rabies (R)
  - ○ Distemper, hepatitis, parvovirus, parainfluenza, leptospirosis (SHPPiL4 / DHPPiL4)
  - ○ Other: ____
- Is regular blood work performed on your dog?
  - ○ No
  - ○ Yes: __________ x per year

3. Activity level of your dog

- What housing conditions mainly apply for your dog?
  - Apartment
  - House
  - Fenced yard
- Does your dog qualify as service or working dog?
  - No
  - Yes:
    - Hunting dog
    - Service dog
    - Other: ____
- Does your dog engage in dog sports activities?
  - No
  - Yes
  - If you checked „yes“: What dog sport(s) does your dog engage in?

    ____
  - If you checked „yes“: How many hours **per week** does your dog spend on **dog sports**?
    - < 1h
    - 1h – 2h
    - 2h – 3h
    - 3h – 4h
    - 4h – 5h
    - > 5h, approx. hours per week: ___
- How many hours **per day** (considering an average working day) does your dog spend on **other physical activities** (walking, cycling, hiking, etc.)?
  - < 1h
  - 1h – 2h
  - 2h – 3h
  - > 3h, approx. hours per day: ___________
  - What kind of physical activity per day is involved? [multiple choices can be selected]
    - Leash walks
    - Biking (running alongside the bike)
    - Off-leash walks
    - Jogging
    - Dog park / play area
    - Other: ____________________________________
  - Approximately what distance (kilometers or miles) is covered (on average) through the activities selected above?

    Activity: __________________________ distance: _____________________________

    Activity: __________________________ distance: _____________________________

    Activity: __________________________ distance: _____________________________
- How many hours are spent on **other physical activities** (walks, cycling, hiking, etc.) on **days off-work** (e.g., weekends, bank holidays)?
  - < 1h
  - 1h – 2h
  - 2h – 3h
  - 3h – 4h
  - 4h – 5h
  - > 5h, approx. hours per day:_____

4. Dietary history of your dog

- What reason or indication led to selecting a raw meat-based diet (RMBD) for your dog?

  ____

  ____

  ____

- What sources of information about feeding an RMBD have you utilized?
  - o Friends / family
  - o Other dog owners
  - o Dog training school / dog trainer
  - o Breeder
  - o RMBD shop
  - o Pet shop
  - o Internet
  - o Books / other primary literature
  - o Veterinarian (not specialized in nutrition)
  - o Certified veterinary nutritionist
  - o Other: ____
  - If you checked "internet": Please provide information about the internet forum(s) used:

    ____

- Is this dog your first dog to receive an RMBD?
  - o Yes
  - o No; I have also fed _______ dogs with an RMBD
- For how long have you been feeding your dog an RMBD?

  ____

- Has your dog received an RMBD since puppyhood?
  - o No
  - o Yes; since the age of ______________________________
- How many times per day does your dog receive food?
  - o 1x per day
  - o 2x per day
  - o 3x per day
  - o 4x per day
  - o Other: __________________________________
- What sources are used to obtain the meat for your dog's RMBD?
  - o Local RMBD shop
  - o Pet shop
  - o Local butcher
  - o Online RMBD shop
  - o Local farm
  - o Other: ____________________________
- What condition is the meat for RMBD feeding received?
  - o Fresh
  - o Frozen
  - o Other: ____
- What source(s) is/are used to obtain the fruit(s) / vegetables for your dog's RMBD?
  - o Local RMBD shop
  - o Supermarket / discounter
  - o Online RMBD shop
  - o Other: ____________________________

- Which source of carbohydrates is useed for your dog’s RMBD ration?
  - Boiled noodles
  - Boiled potatoes
  - Boiled rice
  - (Simmered) oats
  - Other: ____
- What dietary supplements, if any, are included in your dog’s RMBD ration?
  - Ready-mix powder(s): ____
  - Self-made powder(s): ____
  - Oil / water solutions: ____
  - Other: ____
  - No supplements are included.
- What sources are used to obtain these feed supplements?
  - Local RMBD shop
  - Pet shop
  - Online RMBD shop
  - Veterinary office
  - Other: _______________________________________
- What kind of treats does your dog receive?
  - Dried meat
  - Commercial RMBD-based treats
  - Home-made treats
  - Other: __________________________________
- What sources of information were utilized to formulate your dog’s RMBD ration?
  - Local RMBD shop
  - Pet shop
  - Internet RMBD-ration calculator
  - Certified veterinary nutritionist
  - Veterinarian (not specialized in nutrition)
  - Other: ______________________
- How do you rate your dog's body condition on a scale from 1 (way too thin, emaciated) to 9 (severely obese), with a score of 5 reflecting at ideal body weight for the dog’s size?

  ____

Your dog’s participation in this study is highly appreciated!
