# Supplementary material for: Lower prevalence of obesity and nutritional imbalances in dogs fed a raw meat-based diet (RMBD) compared to a commercial complete diet
Source: BMC Vet Res. 2026 Feb 6;22:127. doi: 10.1186/s12917-026-05283-4 (PMC12930774; doi:10.1186/s12917-026-05283-4)
Supplement: Supplementary file 7 — Additional file 7. Study questionnaire for CD-feeding dog owners used in the study. [file 12917_2026_5283_MOESM7_ESM.pdf]

Study questionnaire: Dogs receiving complete commercial dog food (CD)

1. Patient information

- Name: ____
- Date of birth: ______________________________________
- Breed: ____
- Sex
  - ○ Male intact ○ Male neutered
  - ○ Female intact ○ Female spayed
- Does your dog have any known medical conditions?
  - ○ No ○ Yes: ____
- Does your dog receive any long-term medication?
  - ○ No ○ Yes: ____
- Does your dog live with partner animals in the same household, especially other dogs?
  - ○ No ○ Yes: ____
  - If you checked “yes”: What diet(s) do the other pets receive?
  - ○ Commercial complete food
    Please provide information about the manufacturer, complete name, and type of food (e.g., wet/canned or dry/kibble food): ___________________________________
  - ○ Raw meat-based diet (RMBD)
  - ○ Other: ____

2. Annual deworming schedule and vaccination protocol

- Deworming schedule – how often is your dog dewormed?
  - ○ 1x per year (every 12 months) ○ 2x per year (every 6 months)
  - ○ 3x per year (every 4 months) ○ 4x per year (every 3 months)
  - ○ Based on the results of routine fecal parasitology examination
  - ○ Other: ____
  - Dewormer (drug) used: ____
- Vaccination protocol – what is your dog vaccinated against?
  - ○ Rabies (R)
  - ○ Distemper, hepatitis, parvovirus, parainfluenza, leptospirosis (SHPPiL4 / DHPPiL4)
  - ○ Other: ____
- Is regular blood work performed on your dog?
  - ○ No
  - ○ Yes: __________ x per year

3. Activity level of your dog

- What housing conditions mainly apply for your dog?
  - ○ Apartment ○ House ○ Fenced yard
- Does your dog qualify as service or working dog?
  - ○ No
  - ○ Yes: ○ Hunting dog ○ Service dog
    ○ Other: ____
- Does your dog engage in dog sports activities?
  - ○ No ○ Yes
  - If you checked „yes“: What dog sport(s) does your dog engage in?

    ____
  - If you checked „yes“: How many hours **per week** does your dog spend on **dog sports**?
  - ○ < 1h ○ 1h – 2h ○ 2h – 3h
  - ○ 3h – 4h ○ 4h – 5h ○ > 5h, approx. hours per week:____
- How many hours **per day** (considering an average working day) does your dog spend on **other physical activities** (walking, cycling, hiking, etc.)?
  - ○ < 1h ○ 1h – 2h ○ 2h – 3h
  - ○ > 3h, approx. hours per day: ___________
  - What kind of physical activity per day is involved? [multiple choices can be selected]
  - ○ Leash walks ○ Biking (running alongside the bike)
  - ○ Off-leash walks ○ Jogging
  - ○ Dog park / play area ○ Other: ___________________________________
  - Approximately what distance (kilometers or miles) is covered (on average) through the activities selected above?

    Activity: __________________________ distance: _____________________________

    Activity: __________________________ distance: _____________________________

    Activity: __________________________ distance: _____________________________
- How many hours are spent on **other physical activities** (walks, cycling, hiking, etc.) on **days off-work** (e.g., weekends, bank holidays)?
  - ○ < 1h ○ 1h – 2h ○ 2h – 3h
  - ○ 3h – 4h ○ 4h – 5h ○ > 5h, approx. hours per day:_____

4. Dietary history of your dog

- What sources of information about dog feeding practices have you utilized?
    - o Friends / family
    - o Other dog owners
    - o Dog training school / dog trainer
    - o Breeder
    - o RMBD shop
    - o Pet shop
    - o Internet
    - o Books / other primary literature
    - o Veterinarian (not specialized in nutrition)
    - o Certified veterinary nutritionist
    - o Other: ____
- If you checked “internet”: Please provide information about the internet forum(s) used:

  ____

- How many times per day does your dog receive food?
    - o 1x per day
    - o 2x per day
    - o 3x per day
    - o 4x per day
    - o Other: _____________________________________
- What type of dog food do you feed your dog?
    - o Wet complete commercial dog food (canned)
    - o Dry complete commercial dog food (kibble)
    - o Both: wet and dry complete commercial dog food
- What sources are used to obtain the complete commercial dog food?
    - o Pet shop
    - o Online shop
    - o Veterinary office
    - o Other: _____________________________________
- Please state the manufacturer and name of the complete commercial dog food you feed (*for example: Royal Canin „Gastro-Intestinal“*):

  ____

- What types of meat are included in the complete commercial dog food you feed your dog? [multiple choices can be selected]
    - o Poultry meat
    - o Beef
    - o Veal
    - o Pork
    - o Horse meat
    - o Mutton meat
    - o Lamb meat
    - o Other: _____________________________________
- What is the composition of the complete commercial dog food your receives?
    - o Percentage of crude protein: ____
    - o Percentage of crude fiber: ____
    - o Other: ____

- How many grams (g) of complete commercial dog food does your dog receive per meal?
  - Wet complete commercial dog food (canned): ________________g/meal
  - Dry complete commercial dog food (canned): _________________g/meal
- What basis was used to determine the amount of food your dog receives?
  - Feeding recommendation as per package label
  - Calculation by owner
  - Calculation by a veterinarian
  - Other:____
- Does your dog regularly receive any other foods beyond the complete commercial dog food? Please do ***not*** list ***treats***.
  - Carrots
  - ____
  - ____
- What kind of treats do you feed your dog?
  - Commercial dog treats
  - Home-made treats
  - Dried meat
  - Other: ___________________________
- How do you rate your dog's body condition on a scale from 1 (way too thin, emaciated) to 9 (severely obese), with a score of 5 reflecting at ideal body weight for the dog’s size?

  ____

Your dog’s participation in this study is highly appreciated!
